# ERK5 Is Required for Tumor Growth and Maintenance Through Regulation of the Extracellular Matrix in Triple Negative Breast Cancer

**DOI:** 10.3389/fonc.2020.01164

**Published:** 2020-08-03

**Authors:** Van T. Hoang, Margarite D. Matossian, Deniz A. Ucar, Steven Elliott, Jacqueline La, Maryl K. Wright, Hope E. Burks, Aaron Perles, Fokhrul Hossain, Connor T. King, Valentino E. Browning, Jacob Bursavich, Fang Fang, Luis Del Valle, Akshita B. Bhatt, Jane E. Cavanaugh, Patrick T. Flaherty, Muralidharan Anbalagan, Brian G. Rowan, Melyssa R. Bratton, Kenneth P. Nephew, Lucio Miele, Bridgette M. Collins-Burow, Elizabeth C. Martin, Matthew E. Burow

**Affiliations:** ^1^Section of Hematology & Medical Oncology, Department of Medicine, Tulane University School of Medicine, New Orleans, LA, United States; ^2^Department of Genetics, Stanley S. Scott Cancer Center, Louisiana State University Health Sciences Center, New Orleans, LA, United States; ^3^Department of Biological and Agricultural Engineering, Louisiana State University, Baton Rouge, LA, United States; ^4^Medical Sciences, School of Medicine, Indiana University Bloomington, Bloomington, IN, United States; ^5^Department of Pathology, Stanley S. Scott Cancer Center, Louisiana State University Health Sciences Center, New Orleans, LA, United States; ^6^Department of Pharmacology, School of Pharmacy, Duquesne University, Pittsburgh, PA, United States; ^7^Department of Medicinal Chemistry, School of Pharmacy, Duquesne University, Pittsburgh, PA, United States; ^8^Department of Structural and Cellular Biology, Tulane University School of Medicine, New Orleans, LA, United States; ^9^Cellular and Molecular Biology Core, Xavier University, New Orleans, LA, United States; ^10^Tulane Cancer Center, New Orleans, LA, United States; ^11^Department of Pharmacology, Tulane University School of Medicine, New Orleans, LA, United States

**Keywords:** ERK5 kinase, triple negative breast cancer (TNBC), clustered regularly interspaced short palindromic repeats (CRISPR) knockout, extracelluar matix, metastasis, epithelial–mesenchymal–transition, cryogenic scanning electron microscopy (cryo-SEM)

## Abstract

Conventional mitogen-activated protein kinase (MAPK) family members regulate diverse cellular processes involved in tumor initiation and progression, yet the role of ERK5 in cancer biology is not fully understood. Triple-negative breast cancer (TNBC) presents a clinical challenge due to the aggressive nature of the disease and a lack of targeted therapies. ERK5 signaling contributes to drug resistance and metastatic progression through distinct mechanisms, including activation of epithelial-to-mesenchymal transition (EMT). More recently a role for ERK5 in regulation of the extracellular matrix (ECM) has been proposed, and here we investigated the necessity of ERK5 in TNBC tumor formation. Depletion of ERK5 expression using the CRISPR/Cas9 system in MDA-MB-231 and Hs-578T cells resulted in loss of mesenchymal features, as observed through gene expression profile and cell morphology, and suppressed TNBC cell migration. *In vivo* xenograft experiments revealed ERK5 knockout disrupted tumor growth kinetics, which was restored using high concentration Matrigel™ and ERK5-ko reduced expression of the angiogenesis marker CD31. These findings implicated a role for ERK5 in the extracellular matrix (ECM) and matrix integrity. RNA-sequencing analyses demonstrated downregulation of matrix-associated genes, integrins, and pro-angiogenic factors in ERK5-ko cells. Tissue decellularization combined with cryo-SEM and interrogation of biomechanical properties revealed that ERK5-ko resulted in loss of key ECM fiber alignment and mechanosensing capabilities in breast cancer xenografts compared to parental wild-type cells. In this study, we identified a novel role for ERK5 in tumor growth kinetics through modulation of the ECM and angiogenesis axis in breast cancer.

## Introduction

Mitogen-activated protein kinases (MAPKs) are serine-threonine kinases that have well-established roles in cellular processes including proliferation, differentiation, and regulation of cell fate, namely survival and apoptosis (1, 2). Downstream effectors of MAPK include transcription factors as well as MAPK-activated protein kinases (3). ERK5, which is activated by MEK5, is distinct from other conventional MAPK family members because it can be autophosphorylated on amino acid residues not conserved in other MAPK members, it is twice the size of other MAPKs, and its unique C-terminal region enables this kinase to increase the transcriptional activity of target proteins (4, 5). Autophosphorylation of a nuclear localization signaling domain on ERK5 allows for nuclear translocation where it associates with, and activates, downstream transcription factors including Sap1, c-FOS, c-Myc, and MEF2 (6, 7). The MEK5/ERK5 cascade is an important mediator of cell proliferation through induction of cell cycle regulators (8). ERK5 knockdown studies using RNA interference (RNAi) or pharmacological inhibition delay cell cycle progression and decreased proliferation in various cancer types and support the involvement of the MEK5/ERK5 pathway in cancer progression (9). Hyperactivation of MEK5 in estrogen receptor positive (ER^+^) breast cancer cells enhanced estrogen-independent tumorigenesis (10). Furthermore, ERK5 is more highly expressed in breast cancer cells and tumor tissue compared to normal breast cells and tissue (11), and has elevated expression in the more clinically aggressive breast cancer subtype, triple negative breast cancer (TNBC), compared to hormone receptor positive breast cancers (10–12).

Triple negative breast cancer (TNBC) is a heterogeneous subtype in which tumors lack ER, progesterone receptor and HER2/Neu amplification and therefore is difficult to treat with targeted therapies. Gene expression profiling analyses have identified signaling pathways integral to TNBC-specific tumor development (13). The role of ERK5 in TNBC is not clearly defined due to contradictory observations, as some studies demonstrated that shRNA-mediated knockdown of ERK5 did not alter growth dynamics of triple-negative breast cancer xenografts (14, 15). However, partial silencing of ERK5 may not be sufficient to exert anti-tumor effects in certain cell lines (9). While we acknowledge and believe that using shRNA knockdown and pharmacologic inhibition studies are crucial to understand a protein/gene function in cellular systems, here we evaluated how deletion of ERK5 affected TNBC. The Clustered Regularly Interspaced Short Palindromic Repeats/CRISPR-associated protein-9 (CRISPR/Cas9) knockout system has been widely adopted for precision genome editing, and it can be a useful tool in delineating the involvement of MEK5/ERK5 in tumor growth. Extensive reviews have described ERK5 signaling in cancer cell proliferation, tumor development and acquisition of a metastatic phenotype, clearly delineating a role for MEK5-ERK5 in these processes, although the mechanisms of these actions are still under investigation (9, 16).

Dysregulated MEK5/ERK5 signaling is associated with increased metastatic risk in various solid cancer types and less favorable survival outcome (9, 17, 18). Molecular inhibition of ERK5 has been shown to decrease metastasis of breast cancer xenografts in multiple studies (9, 14). Conversely, cancer cells overexpressing MEK5 or ERK5 exhibited a migratory and invasive phenotype (19). Epithelial-to-mesenchymal transition (EMT) is an integral part of metastatic progression whereby cells adopt motile and invasive capabilities through loss of epithelial features and acquisition of mesenchymal features. MEK5 signaling has been implicated in the activation of EMT and transcription factors linked to EMT induction (14, 16, 19–21). ERK5 activation is also associated with enhanced tumor-initiating capacity (22), with inhibition of ERK5 abrogating the pro-tumorigenic effects of MEK5 activity in lung cancer cell spheres (17). Furthermore, ERK5 signaling disrupts actin dynamics leading to alterations in cell migration/invasion potential and metastatic dissemination (14, 16, 17). Additional functions of ERK5 have been defined in cytoskeletal remodeling pathways (22–25).

In addition to regulation of cell intrinsic pathways, more recent studies have examined a role for ERK5 in modulating cell extrinsic properties, such as the extracellular matrix (ECM). ERK5 signaling regulates the expression of matrix metalloproteinase (MMP) family members, which degrade the ECM and facilitates cancer cell dissemination (25, 26). The ECM, which comprises a large part of the tumor microenvironment, has critical functions in the development and maintenance of solid tumor growth (27, 28). Dynamic properties of the microenvironment indicate it is in a constant flux of remodeling and homeostasis (27, 29). The ECM has been shown to have integral roles in cancer progression and metastatic potential (30). MAPK family members have been investigated as indirect regulators of the extracellular matrix of some solid cancer types, through growth factor signaling pathways or downstream activation of FAK, CDC42, and other ECM-associated pathways (31, 32). Furthermore, the interactions amongst integrins, proteoglycans and growth factor signaling pathways, including MAPK signaling, have important roles in maintenance of the ECM (33). Dynamic properties of the ECM can also activate MAPK signaling pathways. Substrate adhesion to integrins can activate Rho, subsequently activating downstream MAPK signaling and stimulating cell proliferation (34, 35). Mechanical force imposed on cells activates growth factor signaling, even in the absence of ligand binding, and integrin-mediated clustering at the fibroblast cell surface vastly amplifies MAPK-mediated growth factor pathways (36). These studies focus on ERK1/2 family members and signaling pathways. However, the direct function of ERK5 in maintenance of the ECM in breast cancer is unknown.

Previous studies in our laboratory have implicated MEK5 signaling in the activation of EMT in TNBC (13). ERK5 expression is elevated in breast carcinomas (13) and ERK5 activation promotes tumorigenesis and metastasis (13). Here, we used the CRISPR-Cas9 system to knock out ERK5 expression in biologically aggressive TNBC cell lines. After confirming reversal of the EMT phenotype consistent with other studies, our main objective in this project was to examine effects of ERK5 deletion on ECM regulation and function. We hypothesized that ERK5 promotes tumor formation and TNBC cell invasion and metastasis through its activity in downstream matrix-associated signaling pathways that are responsible for maintenance of the tumor microenvironment.

## Materials and Methods

### Cells and Reagents

Breast cancer cell lines MDA-MB-231 and Hs-578T were acquired from American Type Culture Collection (ATCC). Liquid nitrogen stocks were made upon receipt and maintained until the start of each study. Cells were used for no more than 6 months after being thawed. Cell lines were authenticated by STR profiling in May 2019. Cells were cultured in Dulbecco's Modified Eagle Medium (DMEM; pH 7.4; Invitrogen, Carlsbad, CA) supplemented with 10% Fetal Bovine Serum (FBS; Hyclone, Salt Lake City, UT), 1% non-essential amino acids, minimal essential amino acids, sodium pyruvate, antibiotic/anti-mycotic and insulin under mycoplasma-free conditions at 37°C in humidified 5% CO_2_ and 95% air. For charcoal-stripped experiments, cells were maintained for 48 h in phenol red-free DMEM without glutamine (Invitrogen, Carlsbad, CA) supplemented with 5% charcoal-stripped FBS (Atlanta Biologicals, Flowery Branch, GA), 1% non-essential amino acids, minimal essential amino acids, sodium pyruvate, pen strep and GlutaMAX. Dimethylsulfoxide (DMSO) was purchased from Fisher Scientific (Waltham, MA).

### Generation of Stable Knockout Cell Lines

Using a pU6-driven guide strand with dual expression cassettes for Cas9/EGFP plasmids-based approach (Horizon, Cambridge, UK), TNBC (MDA-MB-231, HS578T, MDA-MB-157, BT-549) cells were transfected with 5 individual guide strands targeting exons 3 and 5 of the *MAPK*7 (*ERK5*) gene. After transfection, MDA-MB-157 and BT-549 cells were not viable, suggesting in those cell lines ERK5 is required for cell survival. MDA-MB-231 cells, passage 33, was used to generate ERK5-ko cell lines. Hs-589T cells, passage 70, was used to generate ERK5-ko cell lines. After 24 h, cells were sorted for GFP expression using the Becton Dickinson FACSVantage. Flow profiles were analyzed by FACSDiVa software (BD Biosciences). Single cells were plated in a 96-well plate to generate clones. Only one clone in the MDA-MB-231-ERK5-ko cells survived FACS sorting, and six clones were plated after FACS sorting for the Hs-578T-ERK5-ko cells. Clones were propagated and confirmed for ERK5-knockout using qRT-PCR and Western blot. Out of the Hs-578T-ERK5*-*ko clones, clones 3 and 4 had lowest ERK5 protein expression (relative band density <0.3 normalized to parental cell line, which was set to 1). Stable knockout cell lines were tested and authenticated using STR profiling in May 2019. Additionally, MDA-MB-231 cells were stably transfected with pIRES-vector plasmid (Addgene plasmid 10821, Cambridge, MA, USA) with Lipofectamine 2000 per manufacturer's protocol (Invitrogen, Grand Isles, NY, USA) as a control to compare tumors that formed in the GFP-tagged CRISPR/Cas9 ERK5-ko injected cell group. Plasmid (5 μg) was added to serum free opti-MEM (Cat. No. 31985, ThermoFisher Scientific, Waltham, MA) followed by Lipofectamine; after a short incubation period (30 min) the mixture was added to plated MDA-MB-231 cells. After 24 h, pIRES-transfected cells were treated with neomycin (200 ng/mL). Cells were grown in 10% DMEM and treated with neomycin every other day for 2 weeks until only GFP-positive cells remained, which was confirmed using fluorescent microscopy.

### Crystal Violet Staining for Cell Morphology

Cells were plated at a density of 5,000–10,000 cells, depending on average cell size, per well in a 96-well plate in 10% FBS DMEM media and allowed to adhere overnight at 37°C in humidified 5% CO_2_. Plates were harvested, fixed with glutaraldehyde, and stained with 1% crystal violet in 10% methanol solution. Morphological changes were observed using an inverted microscope to visualize with brightfield microscopy.

### Transwell Migration Assay

MDA-MB-231-ERK5-ko, Hs-578T-ERK5-ko and parental control cells (2.5 × 10^4^ cells) were first cultured in charcoal-stripped medium for at least 24 h to remove factors that may affect basal signaling. Then, cells were suspended in 500 μL Opti-MEM and seeded in the upper chamber of a 24-well transwell chamber. Opti-MEM media, which is serum-free, was used as FBS serum was used as the chemoattractant in these experiments. DMEM media containing 10% FBS was added to the lower wells. After 24 h, inner membranes were scrubbed to remove non-migrated cells. Cells on the outer membranes were fixed in formalin and stained with crystal violet. Membranes were excised from the transwell insert and mounted on glass slides. Number of migrated cells were visualized by microscopy and counted. Bars represent percent control migrated cells per 200× field of view ± SEM for triplicate experiments.

### Immunofluorescence Staining

Cells were seeded in 96-well plates at a density of 2,000 cells per well. Cells were fixed in formalin (10% buffered formalin phosphate, Fischer Scientific, Hampton NH), permeabilized with 0.5% Triton-X100 (MP Biomedicals, St. Ana CA) and blocked for 1 h in 0.1% Bovine Serum Albumin + PBS. For proliferation studies, cells were stained with a primary conjugated antibody against Ki-67 (BD Biosciences, Catalog number 558617; 1:200, San Jose CA). For morphology analysis, cells were stained with AlexaFluor® 555 Phalloidin (Cell Signaling, Catalog number 8953; clone 8953, 1:200, Danvers MA) to label actin filaments. For both proliferation and morphology analyses cells were counterstained with DAPI (NucBlue Fixed Cell Stain ReadyProbe, Life Technologies, Carlsbad CA). ApoTome (commercial structure illumination microscopy by Zeiss, Thornwood, NY) fluorescent images were captured on an inverted microscope (Zeiss) and digitally filtered to obtain optical slices. Five images per well were captured at 400×, *n* = 3. For Ki-67 staining, quantified results are represented as percent positive Ki-67 staining (red) out of total number of cells as visualized using DAPI nuclear stain (blue).

### Morphometric Quantification

MDA-MB-231 and Hs-578T phalloidin-stained parental and ERK5-ko cells were utilized for morphometric quantification. In the Aperio Scope program, cell length and width, and cell perimeter were measured and recorded of individual cells. Only cells with the entire perimeter clearly displayed were measured. *N* = 70 cells for MDA-MB-231 parental; *N* = 163 cells for MDA-MB-231-ERK5-ko; *N* = 25 cells for Hs-578T parental; *N* = 27 cells for Hs-578T-ERK5-ko. Cell circularity, aspect ratio (length:width ratio) and overall areas were quantified, and graphically represented. An unpaired *t*-test was performed to evaluate if findings were statistically different between ERK5-ko and parental controls.

(1) $$\begin{array}{r} c=4\pi \left(\frac{A}{P^{2}}\right) \end{array}$$

Equation (1). Circularity Calculation. *c* = cell circularity; π = 3.14, *A* = cell area, *p* = cell perimeter.

### qRT-PCR

Cells were grown in phenol red-free DMEM supplemented with 5% charcoal-stripped (CS) fetal bovine serum (5% CS-DMEM) for 24 h. To determine baseline gene expression, cells were cultured in charcoal-stripped medium for at least 24 h to remove factors that may affect basal signaling. Cells were collected, and total RNA was extracted using the Quick RNA Mini Prep Kit in accordance with the manufacturer's protocol (Zymo Research, Irvine, CA). The quality and concentration of RNA were determined spectrophotometrically by absorbance at 260 and 280 nm using the NanoDrop ND-1000. Total RNA (1 μg) was reverse-transcribed using the iScript kit (BioRad, Hercules, CA) and qPCR was performed using SYBR-green (Bio-Rad Laboratories, Hercules, CA). Cycle numbers of ERK5-ko cells were normalized to β-actin and parental control cells scaled to 1, *n* = 3. For patient-derived xenografts, RNA was isolated from tumor pieces using QIAzol Lysis Reagent (Qiagen, Valencia, CA) and Quick RNA Mini Prep Kit (Zymo Research, Irvine, CA).

### Western Blotting

Cells were cultured in 10% FBS-supplemented DMEM. At confluence cells were collected in PBS, pelleted, and lysed with mammalian protein extraction reagent (MPER) supplemented with 1% protease inhibitor and 1% phosphatase inhibitors (I/II) (Invitrogen, Grand Isles, NY). Samples were centrifuged at 12,000 RPM for 10 min at 4°C to obtain supernatant containing protein extracts. NanoDrop ND-1000 was used to determine protein concentration of samples by absorbance at 260 and 280 nm. After proteins were heat-denatured at 100°C on a heating block, 40 μg of protein was loaded per lane on Bis-Tris-nuPAGE gel (Invitrogen, Grand Isles NY). Protein was then transferred to nitrocellulose membranes using iBlot and iBlot transfer stacks per manufacturer's instructions (Invitrogen, Grand Isles, NY). Membranes were incubated at room temperature with 5% bovine serum albumin (BSA) in 1% Tris-buffered saline, 0.1% Tween 20 (TBS-T) for 1 h to block non-specific binding followed by 4°C incubation overnight with primary antibodies (CDH1: Cell Signaling Technology, Catalog Number 3195; ERK5: Cell Signaling Technology, Catalog Number 3552; p-ERK5: Santa Cruz, Catalog Number 135761). After three 15-min washes in 1% TBS-T, membranes were incubated with appropriate secondary antibodies for at least 1 h. IR-tagged secondary antibodies were purchased from LiCor Biosciences (Lincoln, NE) and used at a 1:10,000 dilution in 5% BSA. Following incubation with secondary antibodies, membranes were washed three times for 15 min per wash in 1% TBS-T, and blots were analyzed by the Odyssey InFRAred Imaging System (LiCor Biosciences). Band density was quantified by LiCor gel imager. Data were normalized to Rho-GDI-α (Santa Cruz Biotechnology, Santa Cruz, CA), serving as loading control (37). Experiments were conducted in triplicate with representative blots shown.

### Proteome Profiler Cytokine Array

A Proteome Profiler Human XL Cytokine Array Kit (R & D Systems, catalog #ARY022B) was used to assess steady-state levels of 105 cytokines in four cell lines: HS78T parental, HS78T-ERK5-ko, MDA-MB-231 parental and MDA-MB-231-ERK5-ko. Briefly, cells were seeded in 10 cm^2^ plates at a density of 60–70% confluence (5–10 × 10^6^ cells) and overnight. The cells were treated with 5% CS FBS and harvested after 24 h. Cells were subsequently lysed and total protein concentration was determined using a BCA assay (Pierce). Each array membrane was blocked according to the manufacturer's protocol and subsequently incubated with 200 μg of total protein from each cell line lysate overnight at 4°C on a rocking platform. The blots were further processed according to the manufacturer's instructions but instead of using the streptavidin-HRP secondary antibody, the blots were incubated with an Alexafluor 488-tagged streptavidin secondary antibody (Life Technologies) followed by imaging on a Versadoc imaging system. Individual dots were quantified using the Versadoc software and normalized to the positive reference controls. Individual cytokines are spotted in duplicate on each membrane; therefore, values shown are the average of the duplicate spots minus background and normalized to reference spots on the individual membranes.

### Whole Transcriptome Sequencing and Pathway Analysis

Hs-578T and MDA-MB-231-ERK5-ko cells were extracted from cell lines for total RNA. Changes in gene expression were determined using next generation sequencing as previously described (38). Analysis of gene changes between MDA-MB-231-ERK5-ko and Hs-578T-ERK5-ko was performed as follows: data sets for comparison where total gene changes between either MDA-MB-231-ERK5-ko vs. MDA-MB-231 parental or HS-578T-ERK5-ko vs. HS-578T parental. For both data sets the following were removed from analysis: small non-coding-lincRNA, miRNA transcripts, 3prime overlapping ncRNA, processed pseudogene, and antisense RNA. Only protein coding RNA transcripts were retained and analyzed. Fold changes were identified as increased if gene fold change compared to parental was above 1.5 and fold gene changes were identified as decreased if below 0.5-fold change. All genes were corrected for false discovery rate (FDR) and were considered significant if adjusted *p*-value was *p* < 0.05. Identification of most significantly altered pathways was performed through David's Bioinformatics Resources v6.8 (https://david.ncifcrf.gov/home.jsp). Gene changes for associated pathways were identified through the gene ontology database.

### Animal Xenograft Studies

Immune-compromised SCID/beige female mice (4–6 weeks old) were obtained from Charles River Laboratories (Wilmington, MA). The animals were allowed a period of adaptation in a sterile and pathogen-free environment with food and water *ad libitum*. Breast cancer cells were collected and viable cells in suspension with 50 μL of sterile PBS mixed with 100 μL Matrigel™ (BD Biosciences, San Jose, CA). For the high concentration Matrigel™ experiments, cells were injected with Matrigel™ that was twice concentrated, containing high amounts of laminin, collagen IV, heparan sulfate proteoglycans and entactin (BD Biosciences, Cat. No. 354248). Injections were made bilaterally into the inguinal mammary fat pads on day 0 (*n* = 5 animals/group) using 27½ gauge sterile syringes. All the procedures in animals were carried out under anesthesia using a mix of isoflurane and oxygen delivered by mask. Tumor size was measured biweekly for 30 days using a digital caliper. Tumor volume was calculated using the following formula: 4/3πLS^2^, where L is the larger radius and S is the smaller radius. At necropsy, animals were sacrificed by CO_2_ exposure followed by cervical dislocation. All procedures involving these animals were conducted in compliance with State and Federal laws, standards of the U.S. Department of Health and Human Services, and guidelines established by the Tulane University Animal Care and Use Committee. The facilities and laboratory animal program of Tulane University are accredited by the Association for the Assessment and Accreditation of Laboratory Animal Care.

### Immunohistochemistry (HC Matrigel Experiment)

After resection, tumors were fixed in 10% buffered formalin for 24 h and embedded in paraffin. Sections of 5 μm in thickness were cut in a microtome and placed on electromagnetically charged slides. Immunohistochemistry was performed using the Avidin-Biotin-Peroxidase complex methodology, according to the manufacturer's instructions (Vector Laboratories, Burlingame, CA). Our modified protocol includes paraffin melting at 58°C in a regular oven for 20 min, deparaffination in xylene, re-hydration through descending grades of alcohol up to water, and non-enzymatic antigen retrieval in 0.01 mol/L sodium citrate buffer, pH 6.0, heated to 95°C for 40 min in a vacuum oven. After a cooling period of 30 min, the slides were rinsed in PBS and treated with 3% H_2_O_2_ in methanol for 25 min to quench endogenous peroxidase. Sections were then blocked with 5% normal goat serum in 0.1% PBS/BSA for 2 h at room temperature. Primary antibodies were incubated overnight at room temperature in a humidifier chamber. Primary antibodies utilized in the present study included a rabbit polyclonal against the endothelial cell marker CD31 (Abcam ab28364, 1:50 dilution), and a rabbit monoclonal anti-E-Cadherin (CDH1, clone EP700Y, ab40772, 1:100 dilution). On the second day, slides were thoroughly rinsed with PBS, and biotinylated secondary anti-rabbit antibodies were incubated for 1 h at room temperature (1:200 dilution). Then sections were incubated with avidin-biotin-peroxidase complexes (Vectastain ABC Elite kit; Vector Laboratories) for 1 h at room temperature, rinsed with PBS, and developed with diaminobenzidine (DAB tablets, Sigma, St. Louis, MO) for 3 min. Finally, the sections were counterstained with Hematoxylin and mounted with Permount (Fisher Scientific, Fair Lawn NJ). For quantification, the number of blood vessels (for CD31 labeling) or positive cells (for CDH1 labeling) were counted in 12 low magnification fields (200×).

### Cardiac Injections

MDA-MB-231 vector parental or MDA-MB-231-ERK5-ko cells were counted (200,000 cells per injection) and suspended in PBS. Immediately after cells were resuspended, injections were initiated to prevent cell aggregation. Mice were anesthetized using isoflurane and oxygen. The chest area was cleaned with alcohol applied with a sterile prep pad. Cells were injected (100 μL per mouse; 5 mice per group) directly into the right atrium using a 26-gauge needle and syringe. A collaborating researcher who has extensive experience with intracardiac injections performed the technique. While brain and bone are the preferred metastatic sites for intracardiac injections, others have shown detectable metastases to the lungs and livers as well (39, 40). While holding the needle at an upwards angle and to the right, the needle was inserted into the second intercostal space, 3 mm to the left of the sternum. The needle was advanced 5 mm and the needle was turned gently until the pulsatile flash of blood was observed entering the hub. The cell suspension was injected over 30 s. Mice were monitored 4 h after the procedure, and biweekly for evidence of distress or illness. After 21 days, mice were euthanized using CO_2_ and cervical dislocation as outlined in the established IACUC protocol from Tulane University. Lungs and livers were harvested and placed in formalin overnight. The organs were paraffin embedded, sectioned, and stained with Hematoxylin and Eosin (H&E) by the core facilities at Tulane University Department of Pathology. Lesions were quantified and areas of lesions were recorded using the Image Scope program.

### Tumor Decellularization

Breast cancer cell line-derived tumors (MDA-MB-231-ERK5-ko and MDA-MB-231-pIRES vector), harvested from the *in vivo* experiment where cells were mixed with normal concentration Matrigel™ and injected in mice, were flash frozen. Then, tumors were thawed for 30–45 min followed by sectioning using an 8-mm biopsy punch (Catalog No. P825; Acuderm Inc., Ft. Lauderdale, FL, USA). Sectioned tumors were decellularized through a protocol modified from Pashos et al. (41). Samples were incubated overnight with PBS at 4°C and then washed with water for 2 h the following day. Next, samples were incubated with a Triton X-100 detergent solution followed by a 2 h water wash. Then, the samples were incubated with a sodium deoxycholate solution (Catalog No. 97062-024; Amresco, Solon, OH, USA) followed by a 2 h water wash. Finally, samples were incubated with a sodium chloride solution for 2 h followed by a 2 h water wash. Samples were stored at 4°C in a PBS solution containing 5× antibiotic/antimycotic until use.

### Cryogenic-Scanning Electron Microscopy Preparation and Imaging

The ECM of decellularized transfected cell-line derived tumors was imaged using scanning electron microscopy (SEM). Samples were fixed in FAA (formaldehyde-acetic acid-ethanol) fixative for 24 h. The samples were rinsed and dehydrated with an increasing series of ethanol, from concentrations 30 to 100%. After the dehydration process, the samples were critical point dried with CO_2_, and then sputter coated with platinum. The prepared SEM samples were then imaged using a FEI Quanta 3D FEG FIG/SEM scanning electron microscope (5 kV).

### Fiber Alignment and Porosity

Fiber alignment and porosity was analyzed through the identification and classification of fiber diameter, orientation and open pores within the region of interest for each SEM image. Samples of each image were analyzed and averaged. The FIJI adaptation feature of ImageJ was used for analysis in combination with an in-house Python script. To identify individual fibers instead of clustering, magnification was kept no lower than 5,000×. Weka Trainable Segmentation (WTS) was used as a plugin for ImageJ to allow for pixel classification based on a machine-learning algorithm. Prior to use the program was trained with previously identified images of fibers identified as “random” or “aligned.” After adjusting the contrast, WTS will used to classify the image as fibrous or non-fibrous. After classifying and segmenting the image with WTS, the FIJI plugins DiameterJ and OrientationJ were used to generate data on the distributions of fiber diameter, orientation, and pore size. To accurately determine fiber diameter, a Euclidean distance transformation algorithm was used to assign a grayscale value to each fibrous pixel equal to that pixel's orthogonal distance to the nearest pore, based on previous methods (42). In the case of fiber intersection, the lines of axial thinning (predicted fiber center-lines) were overlaid onto the Euclidean transformation and all radii values within the grayscale value of the pixel of intersection are subtracted from the centerline. The remaining grayscale values of the centerline are then doubled to produce fiber diameter values. For the accurate detection of pores, an algorithm is used to determine which black pixels constituted as pores. This algorithm, by default, locates clusters of black pixels in the binary image, counts them, and reports the total for each distinct pore detected. In other words, “pores” were defined as areas of non-fiber surrounded by fiber. Pores were excluded if on the image border and a minimum detectable pore size was identified to improve accuracy of the calculation by filtering out small errors in the image segmentation based on previous methods (42). Relative porosity was quantified based on parameters obtained from ImageJ (perimeter, major and minor axis diameters). The total number of pores analyzed were 637 and 500 in 50,000× images and 932 and 902 pores in 25,000× images for control and ERK5-ko decellularized tumors, respectively. One image for each magnification was quantified per group. Representative images reflecting the computer learning methods utilized to quantify porosity and measure relative fiber alignment is found in Supplementary Figure 12C. For the accurate calculation of fiber orientation at each pixel, a related plugin for FIJI called OrientationJ was used. The same axial thinning algorithm described above was used to produce centerlines for each fiber, and each centerline was then enlarged by 2px to reduce error. OrientationJ then calculates the orientation of each pixel by calculating each pixel's Fourier gradient within a 7px Gaussian window. This method is based on evaluating each pixel's structure tensor, of which the largest eigenvector's direction corresponds to the pixel's orientation, based on previously derived methods (43). Probability maps for alignment were generated and individual peaks represent fiber orientation.

### Oscillating Rheometry

Eight-mm samples of decellularized transfected cell-line derived tumors with similar thickness were evaluated for viscoelastic properties. Using a TA Discovery HR-2 rheometer with an 8 mm parallel plate, the storage and loss moduli of decellularized tumors were determined from 0.628 to 62.8 (rad/s) at strain 10% by frequency sweeping.

### Statistical Analysis

Statistical analyses were performed using Graphpad Prism software (Graph-Pad Software, Inc., San Diego, CA). Data were subjected to unpaired Student's *t*-test, with *p*-value < 0.05 considered statistically significant. Studies involving more than two groups were analyzed by one-way analysis of variance (ANOVA) followed by Tukey's *post-hoc* multiple comparison tests. ^*^p < 0.05; ^**^p < 0.01; ^***^p < 0.001. In the porosity quantification experiments (Supplementary Figure 12C), significance was generated from running an unpaired *t*-test with Welch's correction between the data sets. Welch's *t*-test was chosen to compare significant differences between uneven datasets (the inevitable variability in total number of pores quantified in ERK5-ko and control tumors).

## Results

### ERK5 Activity Regulates the EMT Phenotype in TNBC Cells

**Figure 1:**
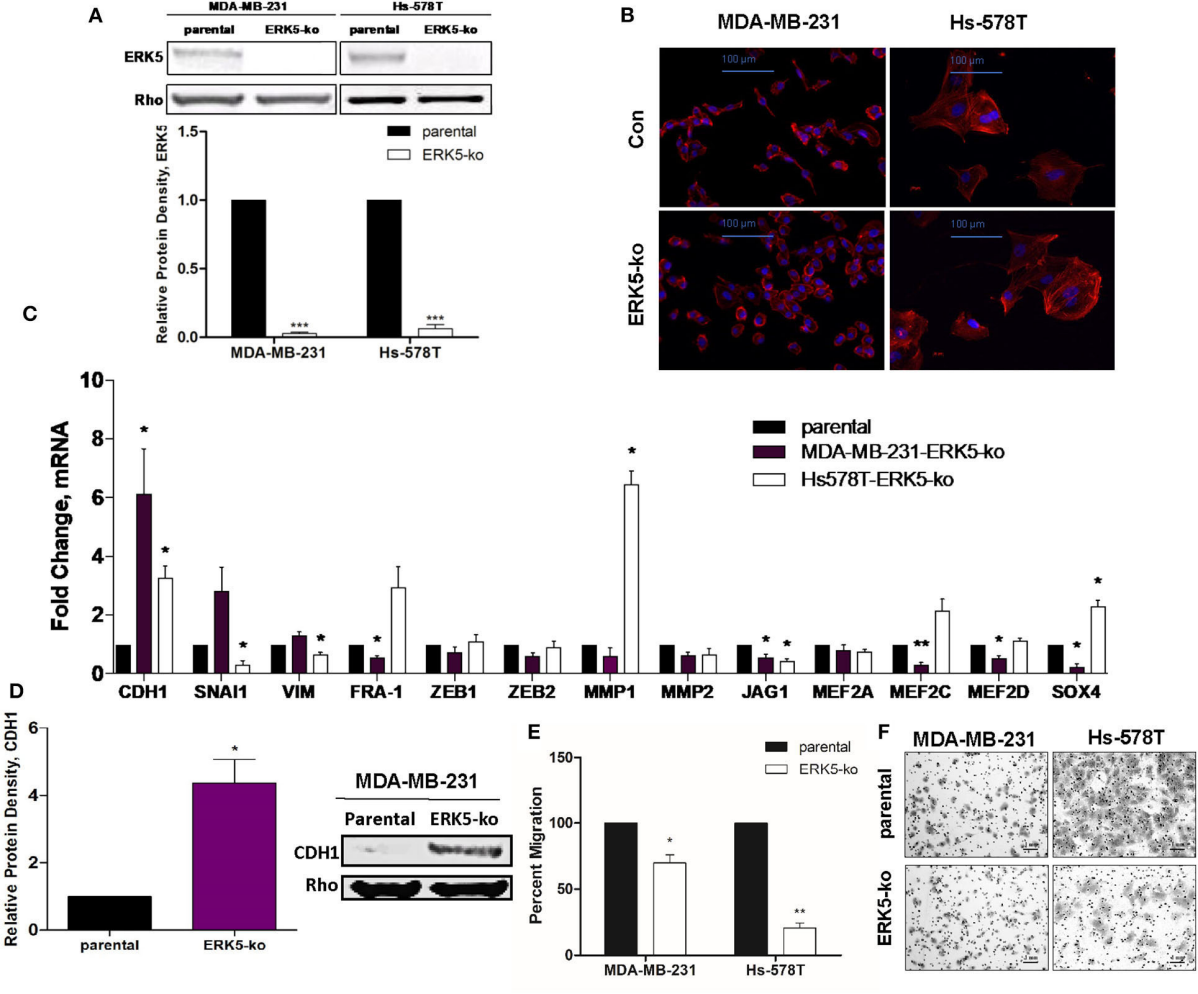
ERK5 ablation promotes an epithelial phenotype in TNBC cells. **(A)** ERK5 expression was knocked out in MDA-MB-231 and Hs-578T cells using the CRISPR/Cas9 system. Total protein was extracted from TNBC-parental and -ERK5-ko cells and Western blot was performed for total ERK5 expression. Rho-GDIα served as a loading control. Bars represent normalized protein density ± SEM with parental control cells set to 1, *n* ≥ 3. **(B)** Representative images of immunofluorescence staining. Phalloidin = red; blue = DAPI nuclear stain; Con = parental control. Representative images are shown at 200× magnification. **(C)** MDA-MB-231- or Hs-578T-parental and -ERK5-ko cells were analyzed for changes in EMT-associated and MEK5/ERK5 downstream gene expression by qRT-PCR. Data was normalized to β-actin and parental controls and samples were run in triplicate. **(D)** Western blot for CDH1 in MDA-MB-231- or Hs-578T-ERK5-ko cells compared to respective parental controls. Data was normalized to Rho-GDIα. *p < 0.05, **p < 0.01. **(E)** Transwell migration assay for MDA-MB-231- and Hs-578T-parental and -ERK5-ko cells. Cells (25,000 cells/chamber) were plated with serum-free media in the top chamber, and 10% FBS media in the bottom chamber as the chemoattractant. Bars represent average number of migrated cells normalized to parental cells ± SEM of triplicate experiments. **(F)** Representative images of the transwell migration assay of MDA-MB-231 and Hs-578T parental and -ERK5-ko cells. Images were captured at 100× magnification and scale bars represent 1 mm.

To assess the role of ERK5 in EMT regulation, we used the CRISPR/Cas9 knock-out approach in phenotypically mesenchymal triple-negative breast cancer cell lines. Expression of ERK5, examined by Western blot, was significantly knocked out in both MDA-MB-231- and Hs-578T-ERK5-ko cells (0.027-fold and 0.063-fold, respectively, *p* < 0.001) compared to parental cells (Figure 1A). Morphological changes induced by ERK5-knockout, from a spindle fibroblast-like shape (characteristic of mesenchymal cells) to a more rounded epithelial phenotype, suggested a partial reversal of EMT (Figure 1B). A representative, population of the cells were sampled and analyzed to quantify the morphologic observations; there was a significant increase in cell circularity (Supplementary Figure 1A), and significant reduction in aspect ratios (Supplementary Figure 1B) in ERK5-ko cells compared to parental controls in both MDA-MB-231 and Hs-578T cells. Overall cell areas were not significantly different between the groups (Supplementary Figure 1C). ERK5 knockout in Hs-578T cells revealed a less dramatic reversal of mesenchymal morphology (Figure 1B). These data support that ERK5 knockout promotes an epithelial cell morphology. Reversal of the mesenchymal phenotype was associated with suppression of mesenchymal-associated gene expressions and upregulation of epithelial genes. MDA-MB-231-ERK5 knockout cells demonstrated suppressed expression of mesenchymal genes (*FRA-1, ZEB1, JAG1*) and of known ERK5 targets (*MEF2A, MEF2C, MEF2D*) (Figure 1C). In Hs-578T-ERK5-ko cells, ERK5-ko suppressed the mesenchymal genes *SNAI1, VIM, JAG1*, and *MMP2* (Figure 1C). These findings that ERK5-ko alters EMT gene expressions in MDA-MB-231 and Hs-578T-ERK5-ko cells compared to parental controls was further demonstrated using RNA sequencing (Supplementary Figure 2). Importantly, we showed that mesenchymal and epithelial gene expressions affected by ERK5-ko are cell line specific. ERK5-ko cells had upregulated E-cadherin (CDH1) transcript (6.13-fold, *p* < 0.05) and protein (4.37-fold, *p* < 0.05) expression in MDA-MB-231 cells (Figure 1D, Supplementary Figure 3). *CDH1* gene expression was upregulated 3.28-fold in Hs578T cells. Consistent with induction of mesenchymal-to-epithelial transition (MET), loss of ERK5 expression decreased transwell motility *in vitro* by 30.2 and 79.4% in MDA-MB-231 and Hs-578T cells, respectively, compared to parental control (Figures 1E,F). Proliferation of MDA-MB-231 TNBC cells, assessed by Ki-67 immunofluorescence staining, was not significantly altered by ERK5 knock-out, suggesting the anti-migratory effects observed were not complicated by differences in proliferative rates (Supplementary Figures 4A,B).

### ERK5 Knockout Impairs Tumor Growth Kinetics and Metastasis *in vivo*

**Figure 2:**
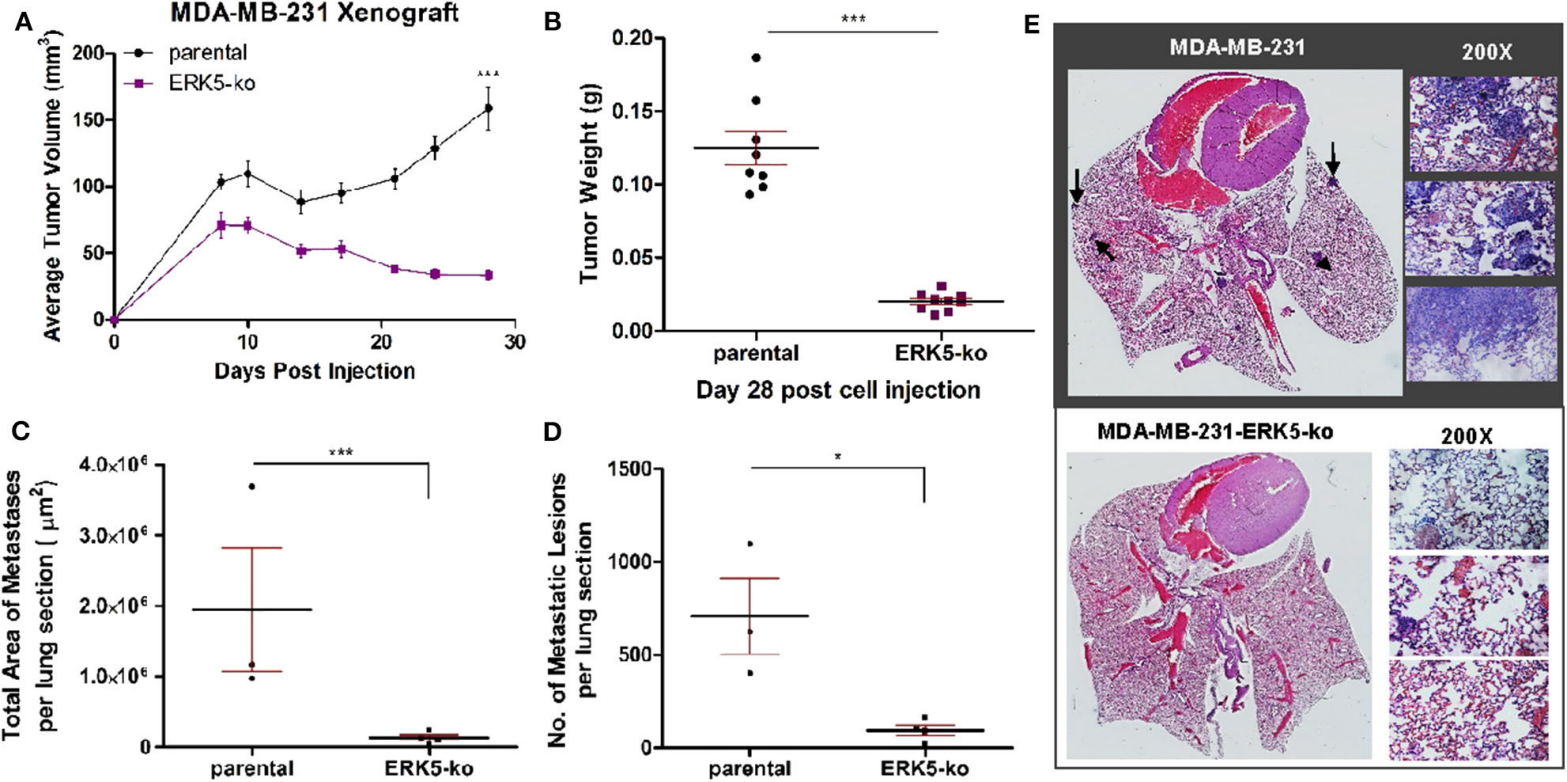
ERK5-ko suppresses tumor growth kinetics and metastasis of MBA-MB-231 cells *in vivo*. **(A)** Female SCID/beige mice (*n* = 5/group) were bilaterally injected into the MFP with MDA-MB-231 vector parental and -ERK5-ko cells. Data points represent mean tumor volume ± SEM. **(B)** At day 28 post-cell injection, primary tumors were excised and weighed; this facilitated distant metastatic seeding. While 10 injections were performed (two injections of cancer cells per mouse), only eight tumors formed in the MDA-MB-231 control group, and nine tumors formed in the MDA-MB-231-ERK5*-*ko group. **(C)** Total area of metastases was quantified using Aperio ImageScope software (Leica Biosystems). Points represent total area of metastases per lung section ± SEM. Parental, *n* = 3; ERK5-ko, *n* = 4. **(D)** Number of metastatic lesions was determined. *p < 0.05, ***p < 0.001. **(E)** H & E staining of lungs 30 days post-survival surgery was used to visualize metastases. Representative lungs from each group demonstrate decreased metastasis in the ERK5 knockout injected animals. Black arrows depict examples of metastases in the lung tissues. Magnified regions were viewed at 200×.

To elucidate the effects of complete CRISPR-Cas9-mediated ERK5 ablation on tumor growth, we employed an orthotopic xenograft model. SCID/Beige mice were inoculated with TNBC parental cell lines (MDA-MB-231, Hs-578T) and ERK5-ko cells into the mammary fat pads. Primary tumor growth kinetics of ERK5-ko cells compared to parental cells was significantly impaired for both tumor size and weight. Tumor weights of the ERK5-ko group was 6.2 times lower than that of MDA-MB-231 parental xenografts (*p* < 0.001) on day 28 post-cell injection (Figures 2A,B). Knockout of ERK5 in Hs-578T cells formed smaller tumors, but due to the very small tumor formation by the parental cells, this was not significant (Supplementary Figure 5A). To determine impact of ERK5 knock-out on the EMT phenotype *in vivo*, CDH1 expression of MDA-MB-231 parental and ERK5-ko primary tumor sections was examined using immunohistochemistry (IHC). While edges of ERK5-ko tumors stained more positively for CDH1 than parental control tumors, no significant change was observed in CDH1 expression between ERK5-ko and parental control tumors overall (Supplementary Figure 6).

Given the prevalence of ERK5 expression in metastatic malignancies (22–24), we then examined the effects of ERK5 knock-out on metastatic progression following surgical resection of the primary tumor on day 28 post-inoculation. Animals were monitored for an additional 30 days, then lungs were harvested for analysis at necropsy. Relative area and number of metastatic foci was reduced in lung sections of MDA-MB-231-ERK5-ko injected mice compared to the parental controls (Figures 2C–E). Micrometastases were detected in mice inoculated with Hs-578T-parental cells and ERK5-ko cells, and the metastases were too small in area for accurate quantification (Supplementary Figure 5B).

Next, using an *in vivo* approach, we analyzed how ERK5 knockout affected colonization capabilities of invasive cells. Using cardiac injections of tumor cells in SCID/Beige mice, we found that while ERK5*-*ko resulted in suppression of tumor growth kinetics and metastasis, ERK5*-*ko cells were still capable of colonizing, suggesting that ERK5 mediates EMT reversal and promotes an epithelial phenotype. MDA-MB-231-ERK5-ko cells increased colonization of lungs in two of the five mice in the ERK5-ko group compared to injected MDA-MB-231 vector parental cells (Supplementary Figures 7A,C). Overall, this data was not significant. Additionally, colonization of the liver did not differ significantly between the two tested groups (Supplementary Figures 7B,D). While these findings suggested the transition to an epithelial phenotype of the ERK5-ko cells allowed for direct metastases to form, extrapolating conclusions from this experiment was limited by the insignificance amongst the data sets.

### ERK5 Alters Tumor Architecture and Extracellular Matrix Composition

**Figure 3:**
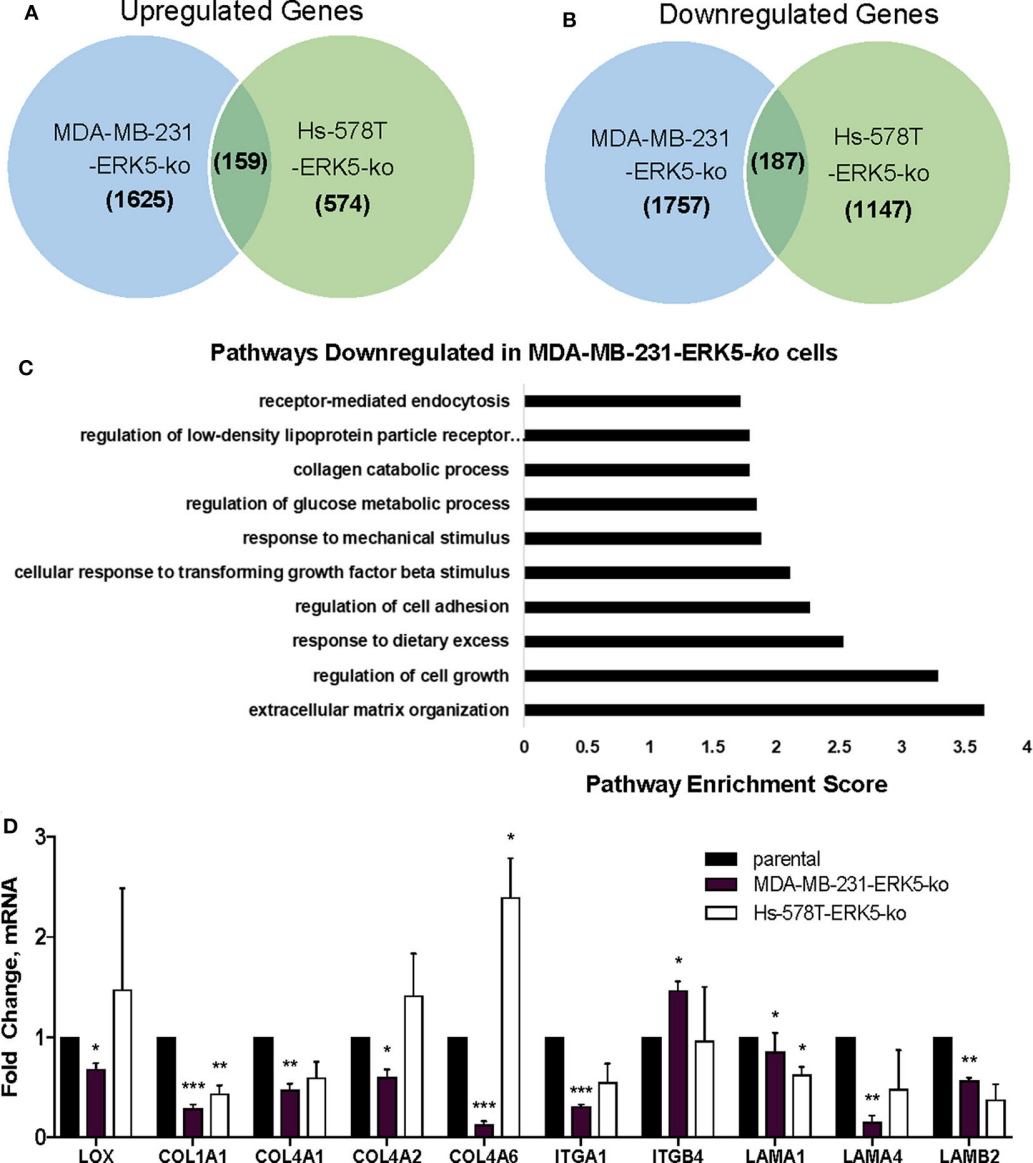
ERK5 deletion results in loss of extracellular matrix components. RNA-sequencing was performed with the MDA-MB-231- and Hs-578T-parental and -ERK5-ko cell lines. Genes were identified that were commonly **(A)** upregulated and **(B)** downregulated in TNBC ERK5-ko cells normalized to parental controls. **(C)** Pathway analysis of the RNA-sequencing showed significant downregulation of signaling pathways that organize the extracellular matrix, regulation of cell growth and regulation of cell adhesion. **(D)** qRT-PCR validation of ECM-associated genes identified from RNA-sequencing. Data was normalized to β-actin and parental controls. Data is represented as averages ± SEM of triplicate experiments. *p < 0.05, **p < 0.01, ***p < 0.001.

Due to the lack of tumor formation in our ERK5-ko cell lines, we next sought to identify potential mechanisms of reduced tumor growth kinetics induced by ERK5-ko using a transcriptomic approach. We performed next-generation RNA-sequencing of MDA-MB-231-ERK5*-*ko and Hs-578T-ERK5-ko cells compared to parental controls to identify key signaling pathways that could regulate tumor formation. ERK5-ko resulted in loss of genes critical for extracellular matrix and mechanosensing signaling and altered pro-inflammatory pathways. The Hs-578T and MDA-MB-231-ERK5*-*ko cell lines only regulated a few shared genes (187 shared upregulated genes and 159 shared downregulated genes) between the cell lines (Figures 3A,B). However, the overall effect of gene regulation led to alterations of the same pathways, with the most commonly downregulated pathways being extracellular matrix organization, regulation of cell growth, regulation of cell adhesion, and response to mechanical stimulation (Figure 3C). The Hs-578T-ERK5*-*ko and MDA-MB-231-ERK5-ko cell lines exhibited decreases in fibrillar and basement membrane associated collagens, fibronectin and select laminins in addition to decreases in the integrins that physiologically interact with these matrix proteins (Supplementary Table I). In MDA-MB-231-ERK5*-*ko cells compared to parental controls, there was downregulation of collagen genes (*COL1A1, COL13A1, COL4A1, COL4A2, COL4A6, COL6A3, COL8A1, COL9A2*), integrins (*ITGA1, ITGA11, ITGA2, ITGA5, ITGA6, ITGB2, ITGB3, ITGB7*), laminins (*LAMA1, LAMB2, LAMB3, LAMC1, LAMC2*) and matrix crosslinking associated genes (lysl oxidase, *LOX*) (Supplementary Table I). When ERK5 expression was transiently transfected in MDA-MB-231-ERK5*-*ko cells, there was restoration of both matrix-associated genes and genes downstream of ERK5 signaling pathways (Supplementary Figure 8). In Hs-578T-ERK5-ko cells compared to parental controls, there was downregulation of *COL3A1, ITGA7*, and *ITGB4*. It should be noted that overall the Hs-578T-ERK5-ko cell line had far less alterations to gene expression than the MDA-MB-231-ERK5-ko cell line. We confirmed using qRT-PCR that ERK5 knockout in both MDA-MB-231 and Hs-578T cells suppresses matrix-associated genes, specifically downregulating *COL1A1, COL4A1*, and *ITGA1*. ERK5 knockout in MDA-MB-231 alone downregulated *COL4A2, COL4A6, LAMA1, LAMA4, LAMB2*, and *LOX* genes (Figure 3D, Supplementary Table II). Importantly, it should be noted that these findings were cell-line specific. Some genes that were downregulated in MDA-MB-231-ERK5*-*ko cells were not downregulated in Hs-578T-ERK5*-*ko, including *LOX*. Evaluation of the RNA-seq data also demonstrated alterations of inflammatory genes associated with the extracellular tumor environment (*IL-8, IGFBP3, ADIPOQ*) in the knockout cell lines. Cytokine analyses of MDA-MB-231-ERK5-ko and Hs-578T-ERK5-ko cells compared to parental controls showed that ERK5-ko resulted in upregulation of *ADIPOQ* and downregulation of *IL-8, OPN, TARC, IGFBP3*, and *MPO* showing a role for ERK5 in regulation of inflammatory signaling pathways in the tumor microenvironment (Supplementary Figure 9). Together this data suggests that ERK5-ko alters the tumor microenvironment through the repression of the cancer secretome. This is observed through the modulation of both paracrine and mechanosensing factors (matrix and integrin).

**Figure 4:**
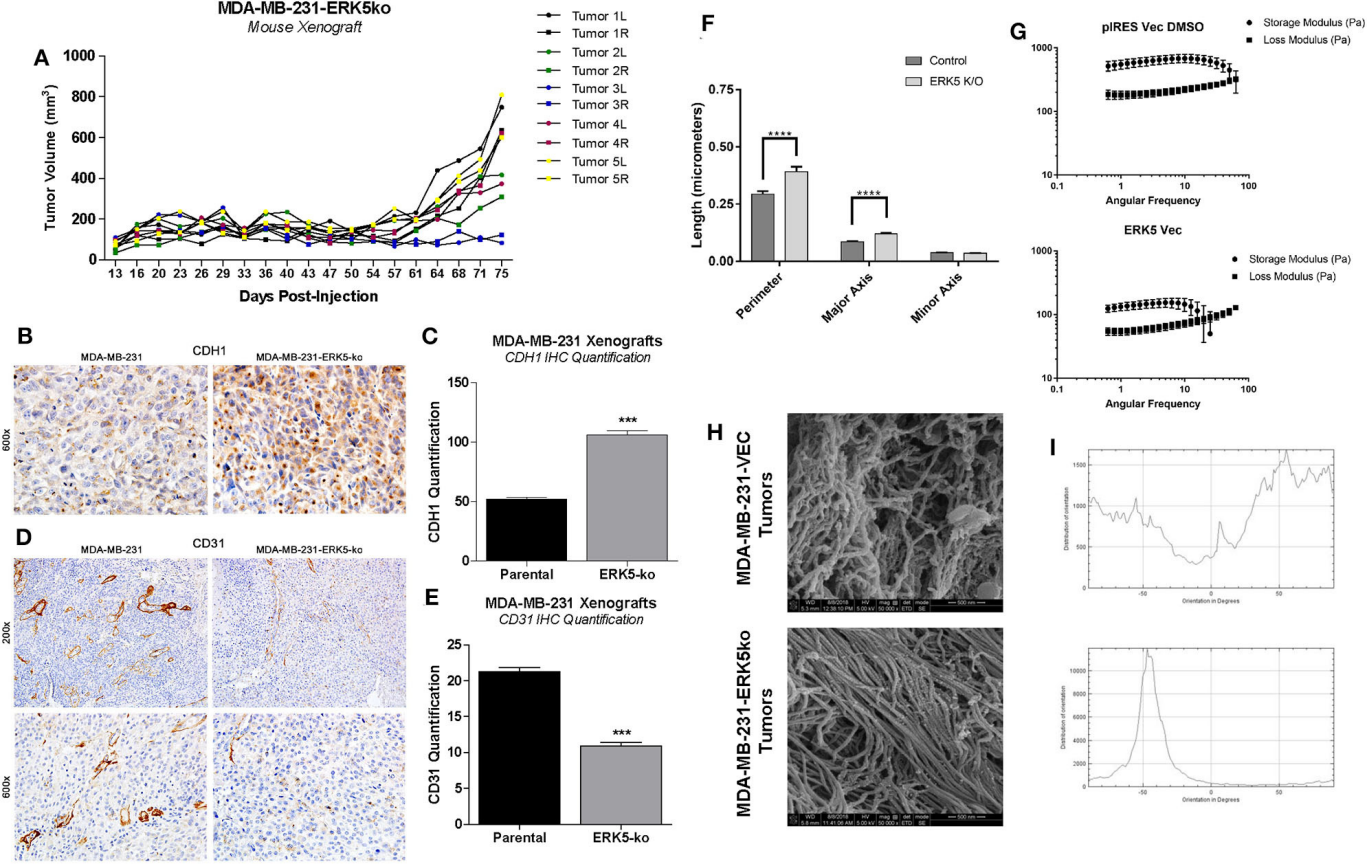
ERK5*-*ko suppresses angiogenesis and increased CDH1 expression of MDA-MB-231 xenografts and require ECM components to restore tumor growth. **(A)** SCID/Beige mice were inoculated bilaterally with MDA-MB-231-ERK5-ko (in vector) cells with high concentration Matrigel™ (HC MG; *n* = 5 mice) in independent experiments (5 × 10^6^ cells/injection). Cells were injected in the mammary fat pads bilaterally and individual tumor growth is represented, with right and left tumors indicated by “R” and “L”, respectively. MDA-MB-231-ERK5-ko tumors formed after a prolonged time period when injected with high concentration Matrigel™. Tumors were measured twice weekly with a digital caliper. Data points represent average tumor volume (mm^3^) ± S.E.M. **(B)** After resection the harvested tumors were formalin fixed, paraffin-embedded and sectioned. Immunohistochemistry for CDH1 performed in xenografts demonstrated a significantly higher expression of E-Cadherin in the cytoplasm of ERK5 knockout tumor cells compared with the MDA-MB-231 parental control cell line. **(C)** The number of CDH1 positive cells was counted in 12 fields of 600× magnification. ***p < 0.001. **(D)** Blood vessels were highlighted by CD31 positive immunohistochemistry staining. **(E)** The number of blood vessels in the ERK5 knockout tumors was significantly lower than their control parental counterparts, evident at both 200× and 600× magnification. **(F)** Quantification of extracellular matrix fibers within tumors derived from MDA-MB-231-ERK5*-*ko or -parental cell xenografts. Relative organization of the aligned fibers is also shown. A computer learning program was trained to distinguish between “fibers” and “non-fibers” based on pixel values; “pores” were defined as regions of non-fiber surrounded by fiber. Relative porosity between the tumor groups were quantified based on measurements of the “pore” regions (perimeter, major and minor axis diameters). The total number of pores analyzed were 637 and 500 in 50,000× images and 932 and 902 pores in 25,000× images for control and ERK5-ko decellularized tumors, respectively. One image for each magnification was quantified per group. ****p < 0.0001. **(G)** Rheometer analyses of decellularized tumors. Storage modulus (Pa) and loss modulus (Pa) are represented in the graphs. Less solid tumors are associated with intersection of storage and loss moduli at a lower angular frequency. **(H)** Representative images of cryogenic scanning electron microscopy of decellularized tumors derived from MDA-MB-231-VEC and -ERK5*-*ko cell lines. Images are shown at 50,000× magnification. **(I)** Relative orientation and alignment of ECM fibers visualized in the scanning electron microscopy images. Orientation is shown on the x-axis (degrees) and distribution of orientation is shown on the y-axis.

To further investigate the role of the tumor microenvironment in tumor growth capabilities of MDA-MB-231-ERK5-ko tumors, we introduced high concentration (HC) Matrigel™ into the xenografts. We bilaterally inoculated mice with MDA-MB-231-ERK5-ko cells combined with HC Matrigel™ (diluted 1:1 with PBS) into the mammary fat pads of female SCID/Beige mice, and recorded tumor volume twice weekly. After a latency period, MDA-MB-231-ERK5-ko tumors formed in all mice (Figure 4A; Supplementary Figure 10). Tumors were harvested and effects of ERK5-ko on cell adhesion and angiogenesis in MDA-MB-231 xenografts were evaluated using CDH1 and CD31 IHC staining, respectively. ERK5-ko xenografts exhibited significantly increased CDH1 expression compared to parental tumors (Figures 4B,C). ERK5-ko significantly suppressed CD31 positivity and thus angiogenesis compared to parental MDA-MB-231 xenografts (Figures 4D,E). Because the ERK5 knockout cell lines had loss of key matrix genes, we next sought to determine if ERK5-ko resulted in loss of tumor architecture. We decellularized the MDA-MB-231 vector parental and knockout tumors harvested from the experiments outlined in Figure 2. Decellularized tumors were then stained with H & E, Masson's trichrome stain and picosirius red to visualize ECM composition between the ERK5 knockout tumors and parental controls. The lack of cellular components in the H & E stain confirmed the tumors were thoroughly decellularized. Masson's trichrome and picosirius red stains to highlight collagen and keratin components did not show any notable differences between the two groups (Supplementary Figure 11).

Cryo-SEM was performed on four randomly selected tumor pieces of the MDA-MB-231 vector and ERK5 knockout tumors. Tumor samples were analyzed for differences in porosity and fiber alignment (Figure 4F, Supplementary Figures 12A,B). To determine how knockout of ERK5 affects tumor matrix microarchitecture, decellularized tumors were evaluated for viscoelasticity using Oscillating rheometry, specifically a frequency sweep (Figure 4G). Both the storage and loss moduli of the control tumor were larger than ERK5-ko tumors at the same angular frequency, implicating tumor stiffness was reduced with ERK5 knockout. In addition, the storage and loss moduli of the ERK5-ko tumors crossed over at an earlier angular frequency than control tumors. This suggests that the architecture of the ERK5-ko tumors was not as connected, or cross-linked, as the control tumors. On average, SEM images taken of parental tumors demonstrated unaligned tumor fibers while the ERK5 knockout tumors had more controlled fiber alignments. This was validated through an adaptation of ImageJ and a machine-learning algorithm to identify fiber alignment. Representative images demonstrating the computer learning techniques utilized to visualize “fibers” and “pores” are shown in Supplementary Figure 12B. Though both tumor types displayed areas of both aligned and unaligned fibers, parental tumors displayed areas of heavily connected fiber formations (Figures 4H,I). SEM imaging of ERK5-ko tumors showed sparse areas of fibrous proteins (Supplementary Figure 12B), suggesting that ERK5-mediated regulation of the ECM might function on a more microscopic level. Additional analysis of fiber alignment and porosity suggests that the ERK5-ko tumors have increased macropore size (Figures 4F,H; Supplementary Figure 12A) and increased alignment of collagen fibers (Figure 4I; Supplementary Figure 12C), which may result in loss of internal tumor architecture observed in the ERK5-ko tumors. Taken together, these data suggest that ERK5-ko results in a loss of tumor extracellular matrix architecture.

## Discussion

Due to the enhanced activity of ERK5 in cancer tissue (10, 44, 45), it has been proposed as a candidate therapeutic target (46). TNBC subtypes have limited targeted therapeutic options, based on lack of expression or overexpression of the commonly targeted proteins, estrogen receptor or HER2 receptor (47). ERK5 is associated with poor distant metastasis-free survival (Supplementary Figure 13A) (48) and has highest expression in clinically aggressive basal B TNBC compared to other breast cancer subtypes (Supplementary Figures 13B,C) (10, 12). The MEK5/ERK5 axis has been extensively studied with respect to epithelial-to-mesenchymal transition and driving a mesenchymal and migratory phenotype (9, 10, 15, 22, 45, 49). Pavan et al. found ERK5 to be a critical gene in breast cancer cell EMT and metastasis using a kinome-wide high content siRNA screen (50). Here, we first confirmed that ERK5 expression regulates the mesenchymal phenotype and cell migration using a CRISPR/Cas9 knockout system. Notably, we observed differences in gene expressions altered by ERK5 knockout in MDA-MB-231 cells compared to Hs-578T cells, suggesting that ERK5 knockout has specific effects in different cell contexts although the cell biology/behavior effects (cell migration, cell morphology) are similar. These findings are consistent with other studies that have shown variable ERK5 expression in cell lines (51, 52). Effects of ERK5 expression loss on metastasis was tested using MDA-MB-231-ERK5*-*ko xenografts. While we observed decreased metastasis to the lungs and livers for the ERK5 knockout group, our experimental design for the *in vivo* experiments did not rule out the possibility that our observations were dually impacted by both direct metastatic effects of remaining secondary tumors and changes in the metastatic capabilities of knockout cells. Future directions in interrogating metastatic effects of ERK5 expression in breast cancer using intracardiac injections would incorporate examining bone and brain metastases (53). Furthermore, consistent with current literature, Hs-578T xenografts are minimally invasive *in vivo* (54), which may explain the lack of contrast in metastatic potential between control and ERK5-ko tumors.

Using CRISPR/Cas9 knockout TNBC cell lines, we then tested the necessity of ERK5 expression in regulation of tumor growth. We discovered cell extrinsic effects of ERK5 deletion on the tumor microenvironment, specifically extracellular matrix composition and fiber alignment. ERK5-deficient TNBC cells have been shown to reduce cell attachment to matrix proteins fibronectin and vitronectin through FAK/PYK2*-*mediated signaling (55). Our data supports these findings, with ERK5 depletion resulting in loss of key extracellular matrix proteins essential for the adherence of integrins (44, 56) through suppressed expression of integrin alpha subunits (ITGA) 1, 2, 10, and 11. Interestingly, we observed reduced expression in the respective collagens to which these integrins bind. This pattern of decreased matrix protein expression with corresponding integrin expression also included: FN1 with ITGA5, and ITGB7 and all laminin subunits (except LAMB2) with ITGA6 (44, 56). Expression of other laminin-binding integrins, -A3B1 and -A7B1, was preserved within these cell lines. High density Matrigel™ may facilitate cell binding and tumor formation by activating these remaining integrin complexes in the ERK5 knockout system.

**Figure 5:**
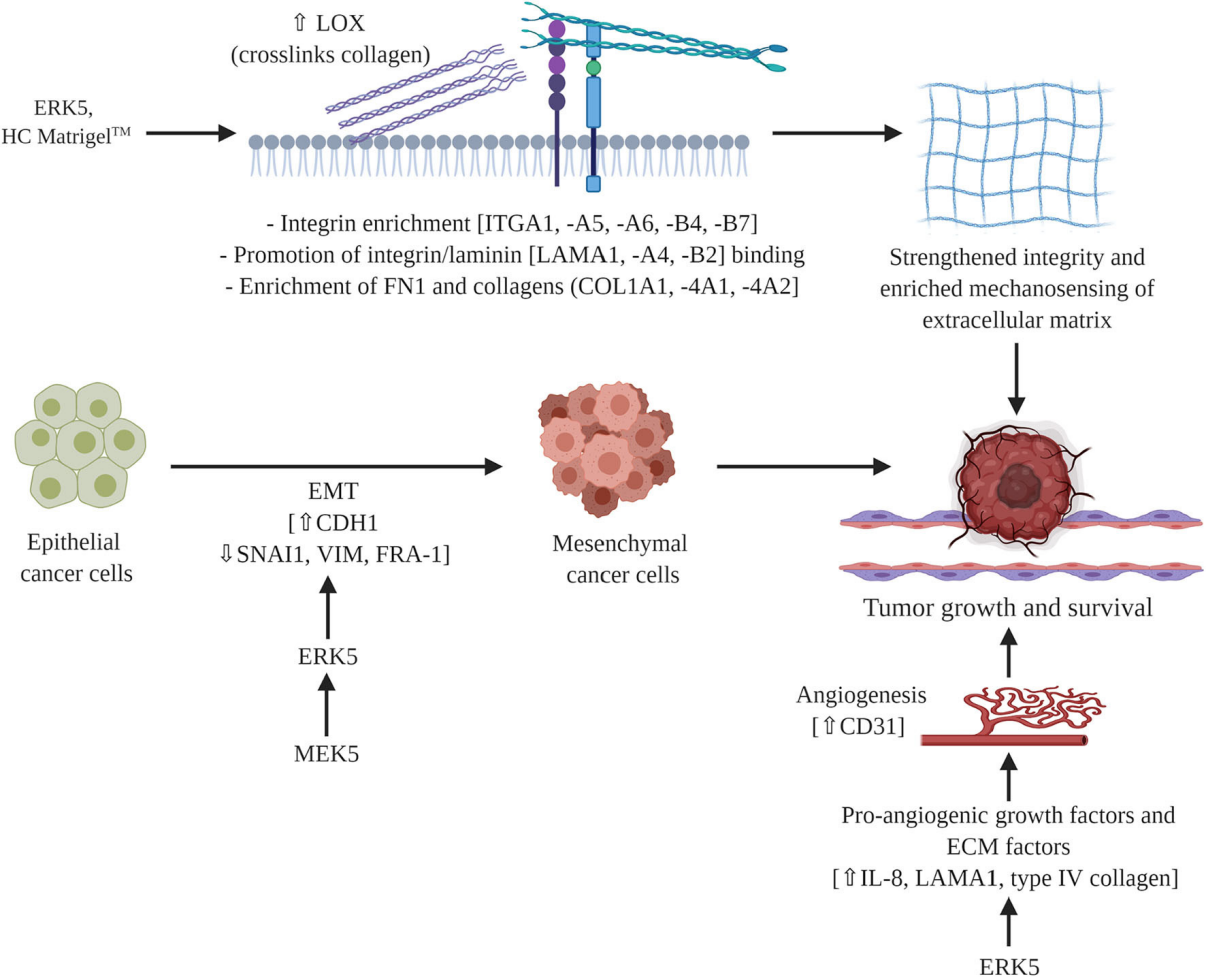
Proposed mechanism of MEK5/ERK5 signaling in triple negative breast cancer. ERK5 mediates breast tumor formation through cell intrinsic and cell extrinsic mechanisms. Pro-angiogenic factors and ECM components converge in a signaling node that is regulated by ERK5 to influence ECM integrity and functionality. Schematic figure was created with BioRender.com.

In this study we uncovered novel mechanisms of ERK5 function through the regulation of matrix structure and angiogenesis. Here we report for the first time that the MEK5-ERK5 axis is necessary in tumor growth and survival by mediating extracellular matrix (ECM) organization, composition, and integrity. We are the first to show mechanistically that ablation of ERK5 expression suppressed ECM genes and secreted cytokines, preventing tumor growth. We propose that this regulation of the ECM axis is both through proliferation-induced mechanosensing and induction of angiogenesis. Our initial studies showed that the angiogenic marker CD31 is lost in ERK5-ko, supporting data from other groups that ERK5 regulates angiogenesis (45). In accordance with this we observed decreased expression in pro-angiogenic cytokines (IL-8) (57) and pro-angiogenic matrix components (laminin, collagens type IV) in our ERK5-ko TNBC system. A dynamic relationship exists between angiogenesis and the ECM in cancer systems that influences tumor behavior (58–60). Pro-angiogenic factors (cytokines, growth factors) affect ECM functionality and integrity (57, 60), and certain ECM factors, including collagen IV, fibronectin, and laminin, are known mediators of vessel formation (57, 58). We propose these pro-stimulatory factors may converge in an ERK5-regulated signaling node to provide the physiologic growth signals necessary to support tumor growth (Figure 5).

In addition to supporting a matrix through integration of MAPK and cytokine pro-angiogenic signaling, our findings indicate a role for ERK5 in matrix mechanosensing and pro-tumor survival through matrix stiffening independent of collagen deposition. Matrix stiffening and mechanosensing have previously been identified to increase TNBC survival. A stiff extracellular matrix has been well-documented as a regulator of cancer progression (61–64). Specifically, stiff breast tissue is a risk factor for development of breast cancer (64, 65) and promotes breast cancer metastasis (66). Increased breast stiffness correlates with an observed increase in fibril collagen (61, 63–65). To the best of our knowledge we are the first to demonstrate ERK5 as a mediator of matrix structure. Our initial analysis of matrix fibers using Masson trichrome does not demonstrates no dramatic change in ERK5-ko tumors compared to parental controls. This is in accordance with our RNA sequencing data that showed only minor changes in expression of main fibrillary collagens and instead identified collagens associated with the basement membrane as being the most significantly altered. After tumors were decellularized the physical properties of the TME could be examined without cellular background influence. We observed larger gaps in the ERK5-ko tumors, suggesting that ERK5 depletion resulted in loss of ECM components that are crucial to tumor formation, specifically focusing on factors that cross-link and strengthen the matrix, such as LOX (Figure 5). Follow-up rheometer analyses show macromolecule degradation in MDA-MB-231-ERK5-ko tumors compared to parental controls. Conducting a frequency sweep on a biological material can reveal the degree of cross-linking that has occurred within that material and, by extension, both the stiffness and viscoelasticity of that material (67). By using a rheometer and increasing the angular frequency of the applied oscillation, the elastic or solid-like component (storage modulus) and the viscous or fluid-like component (loss modulus) of a biological material can be determined. The degree of cross-linking within a biological material can be identified by graphing the results of the frequency sweep. The material is determined to act more fluid-like at the point where the loss modulus overtakes the storage modulus. Therefore, that crossover point would occur at a higher angular frequency if the material has a higher degree of cross-linking. The data in Figures 4F–I suggests that when ERK5 is knocked out, tumors have a lower degree of cross-linking. This could be due to the lower expression of LOX enzymes seen in the ERK5-ko RNA sequencing analysis, a protein that is correlated with metastasis in breast cancer (68). LOX enzymes assist in cross-linking fibril collagens in the ECM; the cross-linking of fibril collagens greatly contributes to overall tumor stiffness. The data on fiber alignment and pore size gained from the machine learning program shows promise as a tool to quickly and objectively evaluate SEM images. The data was consolidated from SEM images taken of the same location of two sets decellularized tumors at two magnifications: 50,000 and 25,000. The program could quickly and accurately quantify both what was and was not evident visually. Quantitatively, the images taken of the ERK5-ko tumors had more aligned fibers than the fibers in the images of the control tumors. In addition, the ERK5-ko tumors displayed larger pore perimeter and major axis when compared to the control. The altered pore size between the ERK5-ko and control tumors may add clarity to the weakened observed structure of the ERK5-ko tumors. To the best of our knowledge we are the first to detail a quantifiable measurement of tumor structure and alignment in decellularized tumors.

This study does have some limitations that should be addressed in future studies. Given the molecular heterogeneity of TNBC (69), ERK5 knockout effects on ECM should be tested in different molecular subtypes of TNBC. Additionally, there was no control for areas within the decellularized samples, so variability between inner and outer tumor axis were not accounted for and should be scrutinized in future studies. This factor may alter tumor alignment and pore size. Furthermore, due to the small size of ERK5-ko tumors, more tumors may have been samples from the outer edges compared to the larger parental tumors. Our data suggest maintenance of tumor architecture may be pivotal in tumor growth and maintenance. In accordance with this, a recent study profiling the effects of tissue porosity and cell-cell contacts demonstrated that increased pore size compared to a hydrogel lead to altered secretome of adipose stem cells (70). We are not aware of any other studies that performed analyses of the effects of tumor porosity and pore size regulation of cancer cell-cell contacts.

These data show a novel role for ERK5 in matrix integrity and subsequent tumor growth kinetics, consistent with a recent study that describes interactions between ERK5 and matrix-associated proteins (71). Mechanistically, we discovered ERK5 has an integral role in regulating the angiogenesis and mechanosensing signaling node (Figure 5). Translational methods utilized in this study to visualize the extracellular matrix introduce new strategies for evaluating potential targets and therapeutics in solid cancers.

## Data Availability Statement

The datasets presented in this study can be found in online repositories. The names of the repository/repositories and accession number(s) can be found below: the NCBI Gene Expression Omnibus (GSE151765).

## Ethics Statement

The animal study was reviewed and approved by Institutional Animal Care and Use Committee, Tulane University School of Medicine. Approval reference number 4299R. Letters of approval are included in Supplementary Materials.

## Author Contributions

VH and MM performed most of the experiments included in this study. VH wrote the first draft of the manuscript. MM completed and formatted the manuscript for submission. SE, JL, HB, AP, DU, and MW contributed to the experiments included in Figure 1 and the mouse *in vivo* experiments. MRB performed the cytokine array experiments. MEB and BC-B generated the original idea of the project and provided funding for the majority of materials and resources needed to complete the experiments. MA and BR performed the IHC experiments included in Supplementary Data. LD performed the IHC experiments and analyses included in Figure 4. AB, JC, and PF contributed to the strategic design of the ERK5-ko metastasis experiments. FF and KN performed the RNA sequencing. LM contributed to the significant revisions in early stages of the manuscript and project design. EM, CK, VB, and JB performed the scanning electron microscopy and biophysical analyses. All authors contributed to the article and approved the submitted version.

## Conflict of Interest

The authors declare that the research was conducted in the absence of any commercial or financial relationships that could be construed as a potential conflict of interest.
