# Supplementary material for: ERK5 Is Required for Tumor Growth and Maintenance Through Regulation of the Extracellular Matrix in Triple Negative Breast Cancer
Source: Front Oncol. 2020 Aug 3;10:1164. doi: 10.3389/fonc.2020.01164 (PMC7416559; doi:10.3389/fonc.2020.01164)
Supplement: Supplementary file 1 [file Data_Sheet_1.DOCX]

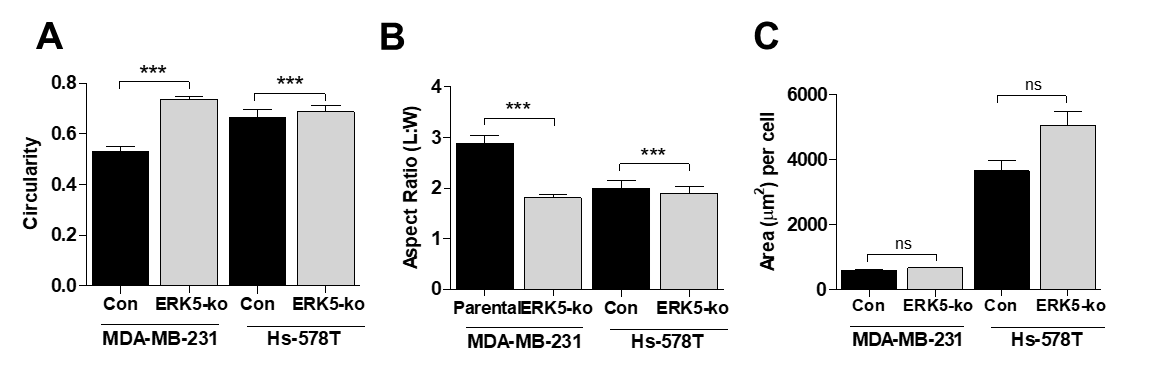


**Supplementary Figure 1. Morphometric quantification of MDA-MB-231 and Hs-578T parental and -ERK5-ko cells based on immunofluorescence staining of cells with phalloidin to highlight actin cytoskeletal filaments.** (A) cell circularity, (B) aspect ratios and (C) overall areas of individual cells were quantified and reported. N = 70 cells for MDA-MB-231 parental; N = 163 cells for MDA-MB-231-ERK5-ko; N = 25 cells for Hs-578T parental; N = 27 cells for Hs-578T-ERK5-ko.
