# Supplementary material for: ERK5 Is Required for Tumor Growth and Maintenance Through Regulation of the Extracellular Matrix in Triple Negative Breast Cancer
Source: Front Oncol. 2020 Aug 3;10:1164. doi: 10.3389/fonc.2020.01164 (PMC7416559; doi:10.3389/fonc.2020.01164)
Supplement: Supplementary file 2 [file Data_Sheet_2.DOCX]

**Supplementary Figure 2.** Epithelial-to-mesenchymal transition associated gene expressions identified from RNA sequencing analyses of MDA-MB-231 and Hs-578T-ERK5*-*ko tumors compared to parental controls.
