# Supplementary material for: ERK5 Is Required for Tumor Growth and Maintenance Through Regulation of the Extracellular Matrix in Triple Negative Breast Cancer
Source: Front Oncol. 2020 Aug 3;10:1164. doi: 10.3389/fonc.2020.01164 (PMC7416559; doi:10.3389/fonc.2020.01164)
Supplement: Supplementary file 3 [file Data_Sheet_3.DOCX]

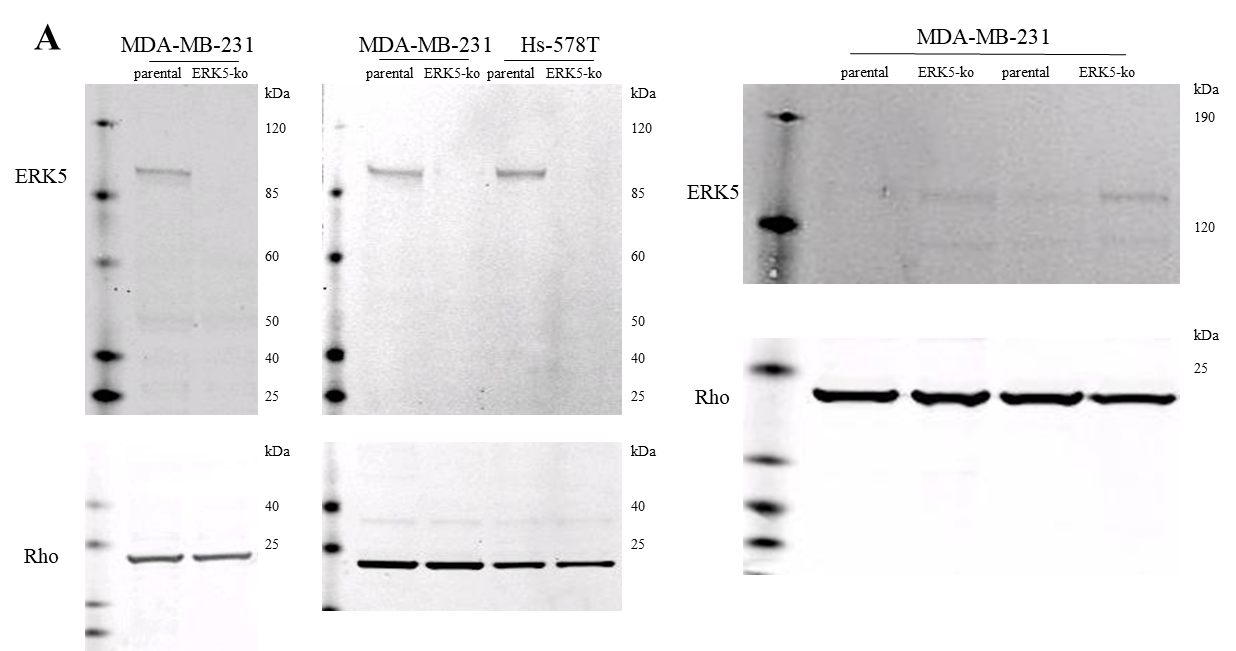


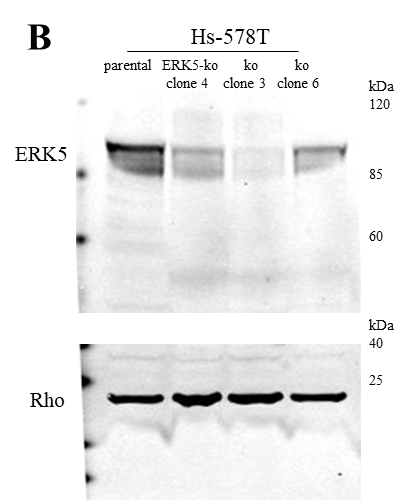


**Supplementary Figure 3.** (A) Full uncropped images of MDA-MB-231 and Hs-578T-ERK5 CRISPR/Cas9 knockout confirmation Western blots. (B) Hs-578T-ERK5 knockout clones. Rho was used for normalization.
