# Supplementary material for: ERK5 Is Required for Tumor Growth and Maintenance Through Regulation of the Extracellular Matrix in Triple Negative Breast Cancer
Source: Front Oncol. 2020 Aug 3;10:1164. doi: 10.3389/fonc.2020.01164 (PMC7416559; doi:10.3389/fonc.2020.01164)
Supplement: Supplementary file 4 [file Data_Sheet_4.DOCX]

**
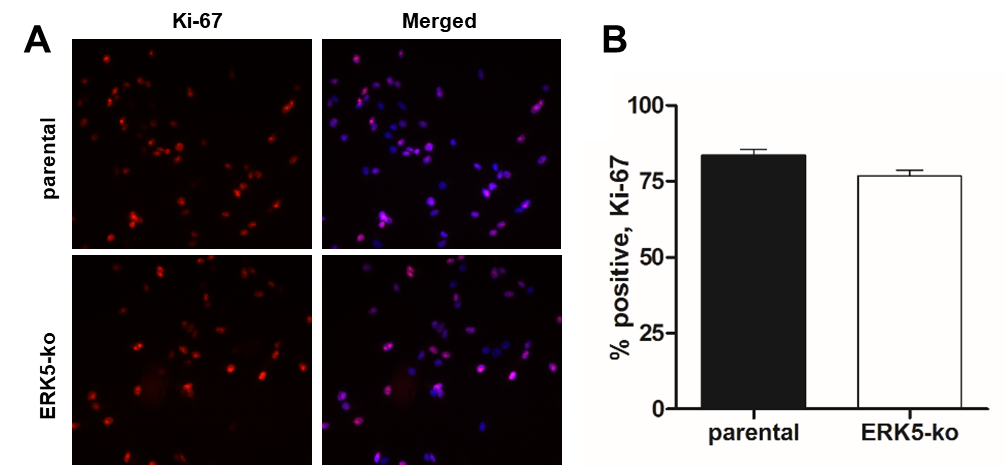
**

**Supplementary Figure 4.** (A) IF (Immunofluorescent) staining of Ki-67 in MDA-MB-231 control and -ERK5-ko cells, viewed at 200x. (B) 5 representative images were taken per well and percentage of Ki-67-positive cells relative to total (DAPI-positive) cells was calculated. Bars represent mean % of Ki-67 positive cells ± SEM of triplicate experiments.
