# Supplementary material for: ERK5 Is Required for Tumor Growth and Maintenance Through Regulation of the Extracellular Matrix in Triple Negative Breast Cancer
Source: Front Oncol. 2020 Aug 3;10:1164. doi: 10.3389/fonc.2020.01164 (PMC7416559; doi:10.3389/fonc.2020.01164)
Supplement: Supplementary file 5 [file Data_Sheet_5.DOCX]

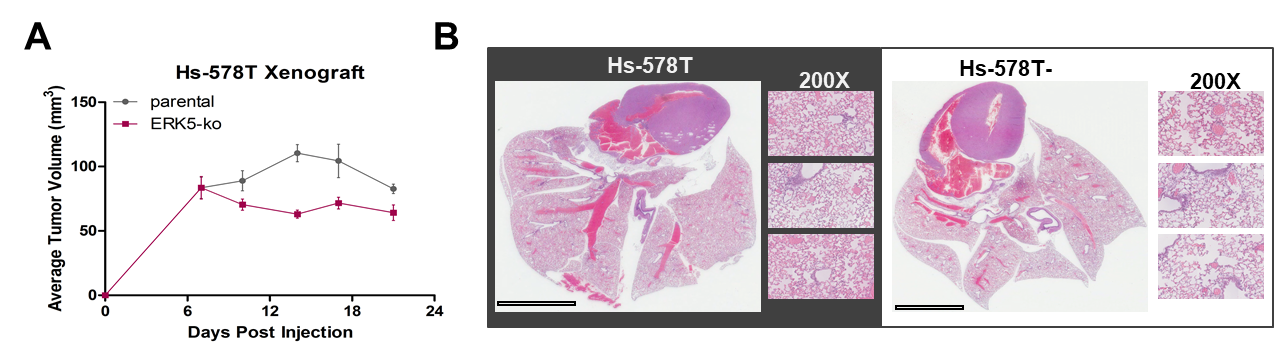


**Supplementary Figure 5. Hs-578T tumor formation in vivo.** (A) Female SCID/beige mice (n = 5/group) were bilaterally injected into the MFP with Hs-578T-parental and -ERK5-ko. Data points represent mean tumor volume ± SEM. (B) At necropsy 30 days post survival surgery, lungs were harvested, fixed and H & E stained. Representative images of lungs from each group, visualized using Aperio ScanScope slide scanner, depict low metastatic potential of Hs-578T tumors. Scale bar = 4 mm; magnified regions were viewed at 200x.
