# Supplementary material for: ERK5 Is Required for Tumor Growth and Maintenance Through Regulation of the Extracellular Matrix in Triple Negative Breast Cancer
Source: Front Oncol. 2020 Aug 3;10:1164. doi: 10.3389/fonc.2020.01164 (PMC7416559; doi:10.3389/fonc.2020.01164)
Supplement: Supplementary file 6 [file Data_Sheet_6.DOCX]

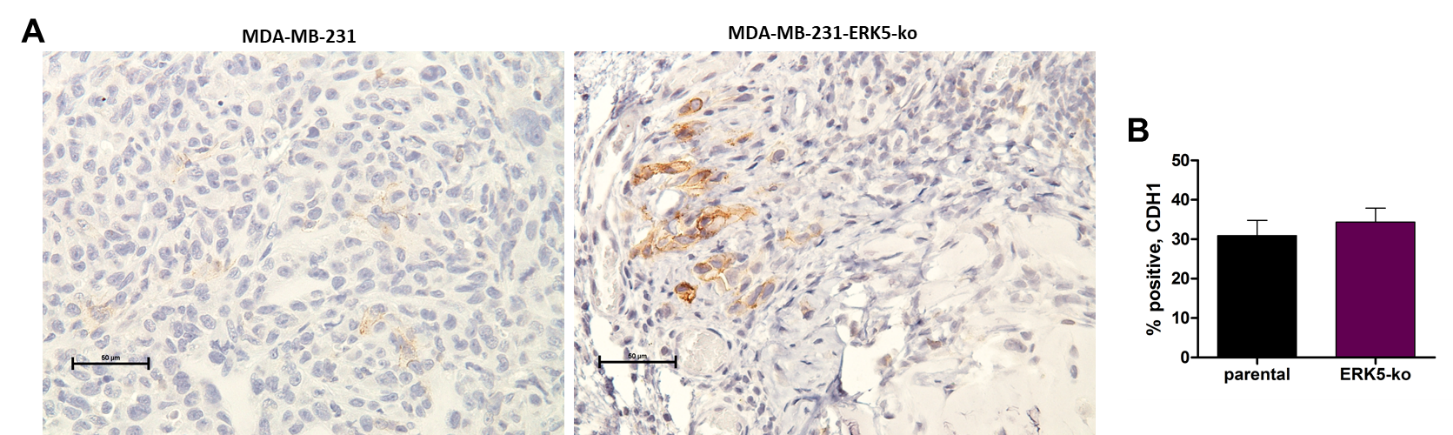


**Supplementary Figure 6. Immunohistochemical staining of CDH1 in MDA-MB-231-parental and -ERK5-ko xenografts.** (A) Tumors were sectioned and stained for CDH1 expression, viewed at 200X. (B) Representative images were taken (5 per tumor section) and percentage of CDH1-positive (stained) cells relative to total number of cells was determined. Bars represent percentage of stained cells per view, n = 3.
