# Supplementary material for: ERK5 Is Required for Tumor Growth and Maintenance Through Regulation of the Extracellular Matrix in Triple Negative Breast Cancer
Source: Front Oncol. 2020 Aug 3;10:1164. doi: 10.3389/fonc.2020.01164 (PMC7416559; doi:10.3389/fonc.2020.01164)
Supplement: Supplementary file 7 [file Data_Sheet_7.DOCX]

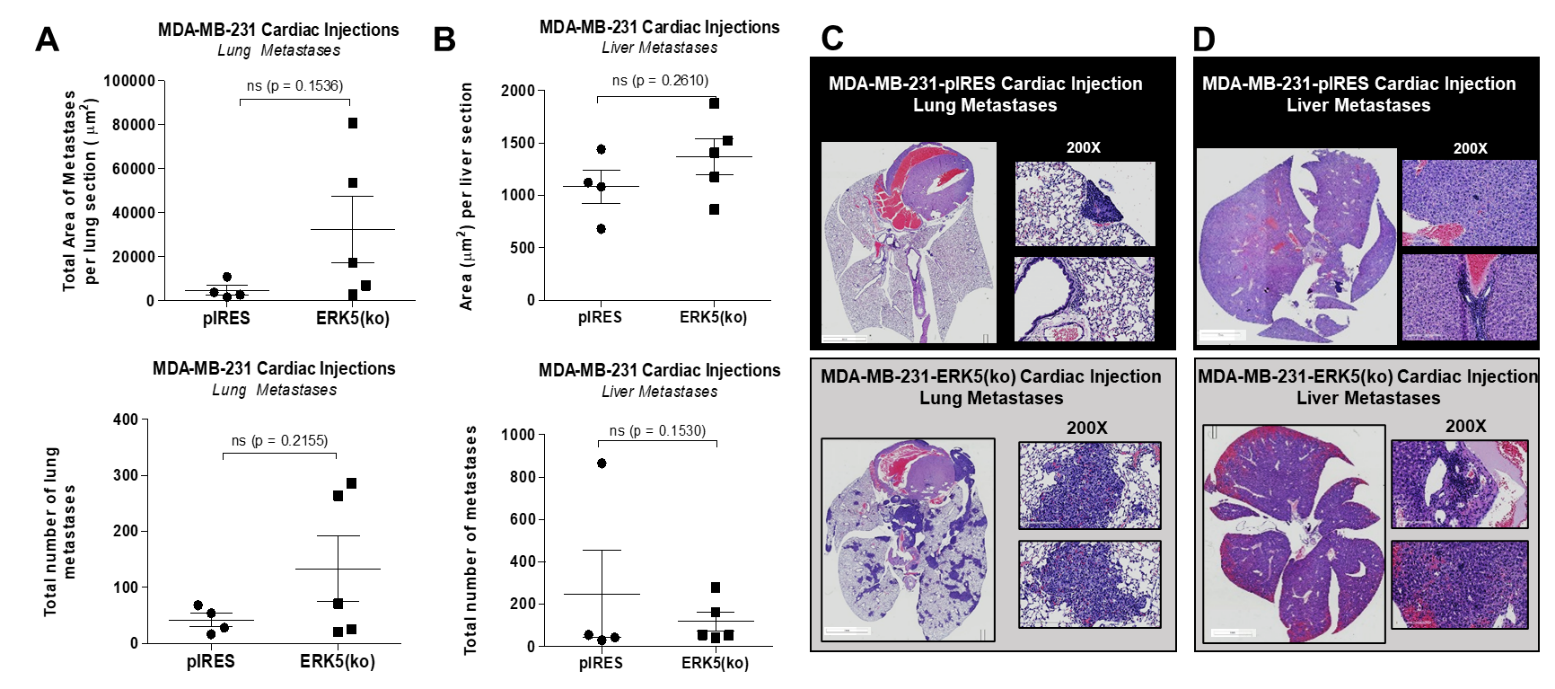


**Supplementary Figure 7. ERK5-ko suppresses tumor growth and metastasis but not colonization of MBA-MB-231 cells in vivo**. Intracardiac injections of MDA-MB-231 vector control and -ERK5-ko cells in SCID/Beige mice was used to evaluate colonization of ERK5-ko cells. For these experiments, a GFP-containing plasmid vector control was utilized to compare to the GFP-expression ERK5-ko cells (data not shown). n = 5/group. (A) ERK5-ko increased the number and area of lung metastatic lesions. (B) The number and area of liver lesions were not affected by ERK5-ko compared to the parental group. *** p<0.001. Representative images of (C) lungs and (D) livers harvested and H & E stained. Inserts are shown at 200x magnification.
