# Supplementary material for: ERK5 Is Required for Tumor Growth and Maintenance Through Regulation of the Extracellular Matrix in Triple Negative Breast Cancer
Source: Front Oncol. 2020 Aug 3;10:1164. doi: 10.3389/fonc.2020.01164 (PMC7416559; doi:10.3389/fonc.2020.01164)
Supplement: Supplementary file 8 [file Data_Sheet_8.DOCX]

**Supplementary Figure 8.** Transient transfection of ERK5 in MDA-MB-231-ERK5ko cells. qRT-PCR for matrix-associated genes (LOX, COL1A1, COL4A1, COL4A2, COL4A6, ITGA1, LAMA4, LAMB1) and genes downstream of the ERK5 signaling pathway (MEF2A, MEF2C, MEF2D, SOX4, JAG1). Data was normalized to β-actin and MDA-MB-231-ERK5-ko cells. None of the data was considered significant when compared to MDA-MB-231-ERK5-ko cells. Error bars represent SEM. All data sets were statistically compared to vector controls using an unpaired t-test, and none of the analyses were statistically significant.
