# Supplementary material for: ERK5 Is Required for Tumor Growth and Maintenance Through Regulation of the Extracellular Matrix in Triple Negative Breast Cancer
Source: Front Oncol. 2020 Aug 3;10:1164. doi: 10.3389/fonc.2020.01164 (PMC7416559; doi:10.3389/fonc.2020.01164)
Supplement: Supplementary file 9 [file Data_Sheet_9.DOCX]

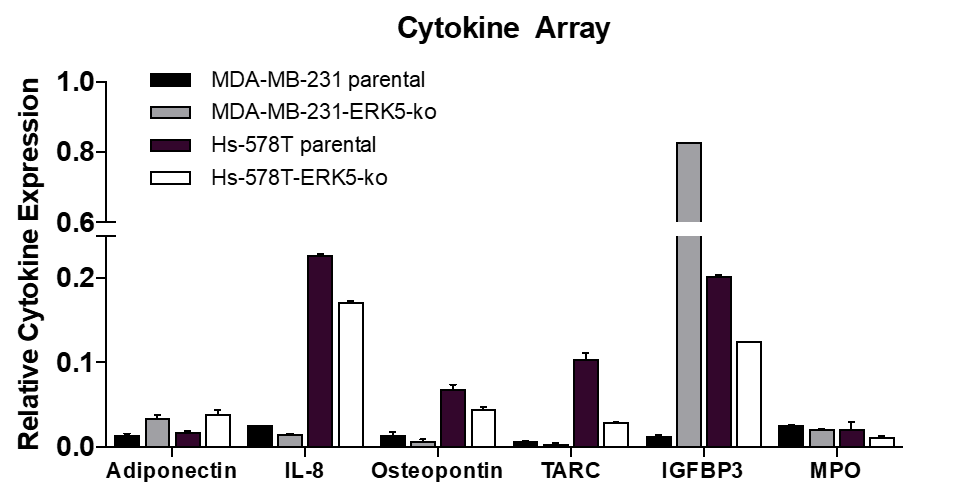


**Supplementary Figure 9. Cytokine array analysis of Hs-578T-ERK5-ko cells.** Relative cytokine expression was analyzed in MDA-MB-231-ERK5ko and HS-578T-ERK5-ko cells compared to parental controls. Error bars represent standard deviation. Samples were run in duplicate in independent experiments.
