# Supplementary material for: ERK5 Is Required for Tumor Growth and Maintenance Through Regulation of the Extracellular Matrix in Triple Negative Breast Cancer
Source: Front Oncol. 2020 Aug 3;10:1164. doi: 10.3389/fonc.2020.01164 (PMC7416559; doi:10.3389/fonc.2020.01164)
Supplement: Supplementary file 10 [file Data_Sheet_10.DOCX]

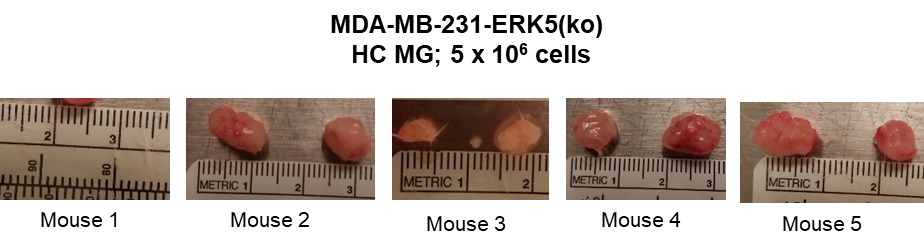


**Supplementary Figure 10.** Representation of tumors that were resected from SCID/Beige mice at the time of survival surgery. MDA-MB-231-ERK5-ko cells were injected with PBS and high concentration Matrigel into the MFPs of mice.
