# Supplementary material for: ERK5 Is Required for Tumor Growth and Maintenance Through Regulation of the Extracellular Matrix in Triple Negative Breast Cancer
Source: Front Oncol. 2020 Aug 3;10:1164. doi: 10.3389/fonc.2020.01164 (PMC7416559; doi:10.3389/fonc.2020.01164)
Supplement: Supplementary file 11 [file Data_Sheet_11.DOCX]

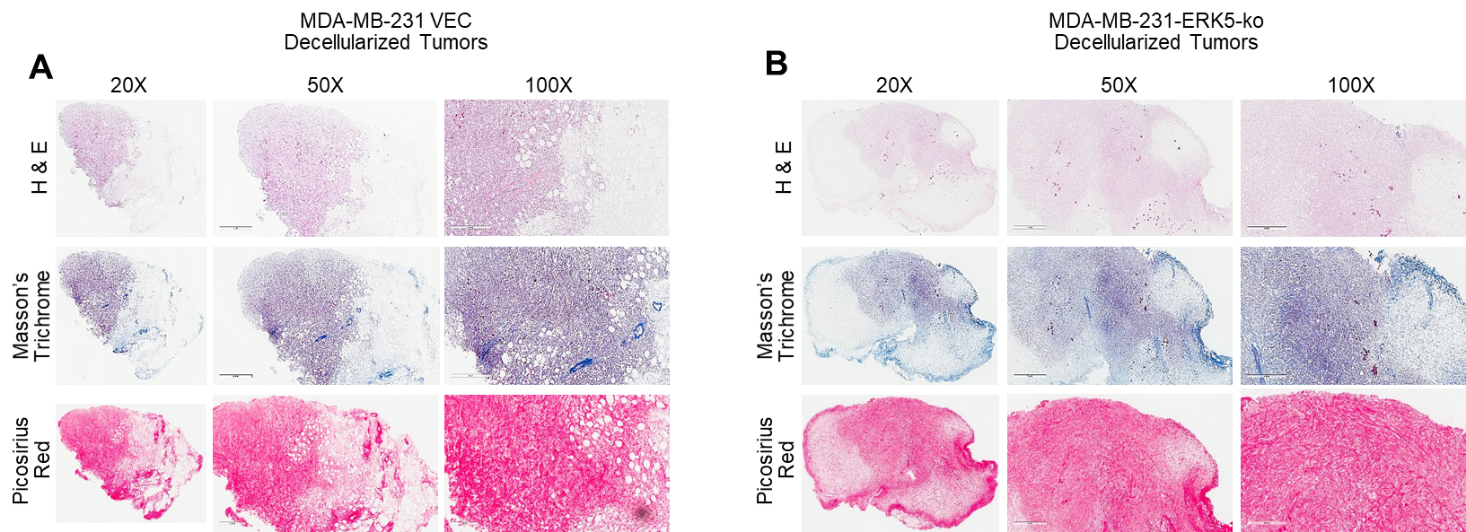


**Supplementary Figure 11.** Decellularized tumors from (A) MDA-MB-231 parental and (B) MDA-MB-231-ERK5-ko xenografts that were used in the cryo-SEM and rheometer analyses. Tumors were formalin fixed, paraffin-embedded and stained with H & E, Masson’s trichrome, or picosirius red. H & E stain was used to demonstrate the lack of cellular components after the decellularization process. Masson’s trichrome stain highlights keratin (red), collagen (blue), and cell nuclei (black), and picosirius red highlights collagens (red). Images are shown at 20X, 50X and 100X magnification.
