# Supplementary material for: ERK5 Is Required for Tumor Growth and Maintenance Through Regulation of the Extracellular Matrix in Triple Negative Breast Cancer
Source: Front Oncol. 2020 Aug 3;10:1164. doi: 10.3389/fonc.2020.01164 (PMC7416559; doi:10.3389/fonc.2020.01164)
Supplement: Supplementary file 12 [file Data_Sheet_12.DOCX]

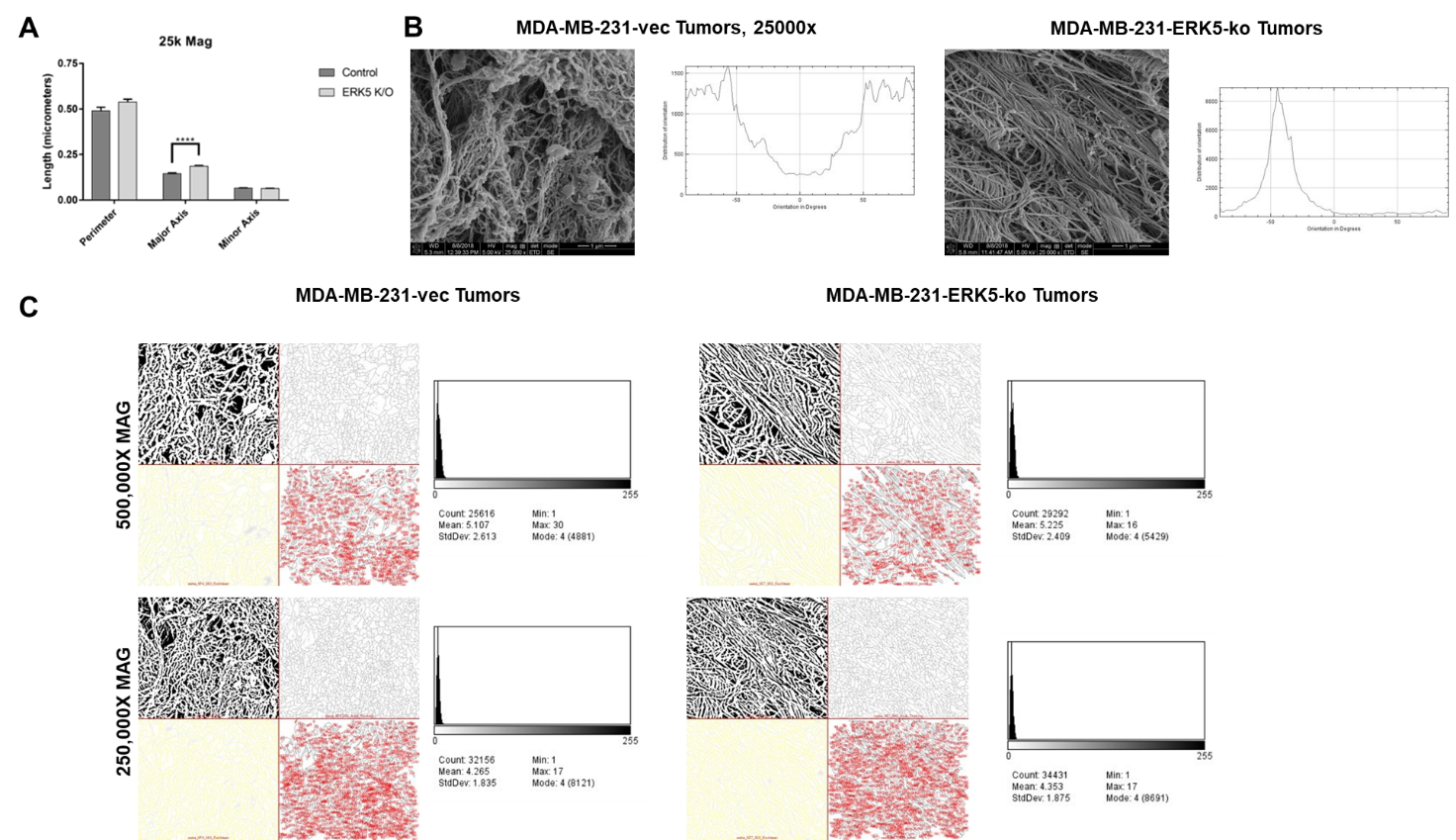


**Supplementary Figure 12.** (A) Quantification of extracellular matrix fibers within decellularized tumors derived from MDA-MB-231-ERK5-ko or -parental xenografts. Based on machine-learning algorithm definitions of ‘fiber’ and ‘porosity’, relative pore perimeter, and long and short axes were quantified. One image for each magnification was quantified per group; measurements of the 25,000X magnification group is displayed. (B) Orientation of matrix fibers in the ERK5-ko and control groups were quantified using cryogenic scanning electron microscopy as outlined in the Methods section. Representative images of cryogenic scanning electron microscopy of decellularized tumors derived from MDA-MB-231-vec and -ERK5-ko cell lines. Images are shown at 25,000X magnification. (C) Representative images of machine learning techniques utilized to quantify porosity parameters. A computer learning program was trained to distinguish between ‘fibers’ and ‘non-fibers’ based on pixel values; ‘pores’ were defined as areas of non-fiber surrounded by fiber. Relative porosity between the tumor groups were quantified based on measurements of the ‘pore’ regions (perimeter, major and minor axis diameters). The total number of pores analyzed were 637 and 500 in 50,000X images and 932 and 902 pores in 25,000X images for control and ERK5-ko decellularized tumors, respectively. ****p < 0.0001.
