# Supplementary material for: ERK5 Is Required for Tumor Growth and Maintenance Through Regulation of the Extracellular Matrix in Triple Negative Breast Cancer
Source: Front Oncol. 2020 Aug 3;10:1164. doi: 10.3389/fonc.2020.01164 (PMC7416559; doi:10.3389/fonc.2020.01164)
Supplement: Supplementary file 13 [file Data_Sheet_13.DOCX]

**
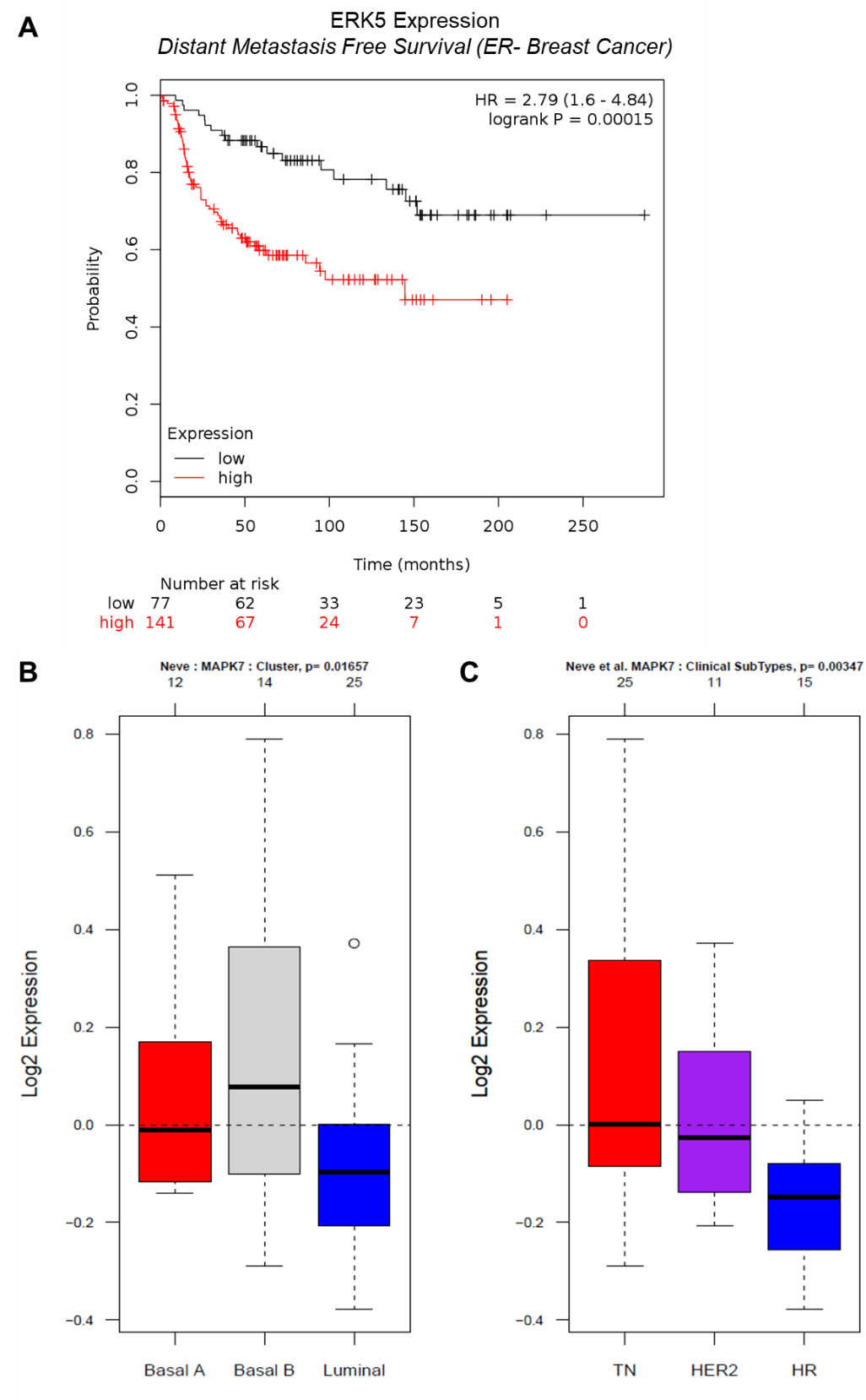
**

**Supplementary Figure 13.** (A) Kaplan-Meier plot of ERK5 expression in estrogen receptor negative (ER^-^) breast cancer. Distant metastasis free survival is shown. High ERK5 expression is associated with reduced survival. Hazard Ratio (HR): 2.79, p = 0.00015. (B) ERK5 (MAPK7) expression is highest in basal B breast cancer subtypes compared to basal A and luminal subtypes. (C) ERK5 expression is highest in TNBC cells compared to HER2-positive or hormone receptor-positive cells. [62]
