# Supplementary material for: ERK5 Is Required for Tumor Growth and Maintenance Through Regulation of the Extracellular Matrix in Triple Negative Breast Cancer
Source: Front Oncol. 2020 Aug 3;10:1164. doi: 10.3389/fonc.2020.01164 (PMC7416559; doi:10.3389/fonc.2020.01164)
Supplement: Supplementary file 14 [file Table_1.DOCX]

**Supplementary Table I. ECM-associated gene expression changes in MDA-MB-231-ERK5-ko and Hs-578T-ERK5-ko cells.** qRT-PCR confirmation of genes identified from RNA sequencing of MDA-MB-231-ERK5-ko and Hs-578T-ERK5-ko cells compared to parental controls. Data is shown as mean ± standard error of mean.

| **Gene** | **MDA-MB-231** | **Hs-578T** |
| --- | --- | --- |
| ***LOX*** | 0.682 ± 0.0575 | 1.48 ± 1.01 |
| ***COL1A1*** | 0.285 ± 0.0424 | 0.433 ± 0.0857 |
| ***COL4A1*** | 0.472 ± 0.155 | 0.597 ± 0.155 |
| ***COL4A2*** | 0.601 ± 0.0767 | 1.42 ± 0.417 |
| ***COL4A6*** | 0.128 ± 0.0321 | 2.40 ± 0.391 |
| ***ITGA1*** | 0.305 ± 0.0204 | 0.553 ± 0.180 |
| ***ITGB4*** | 1.47 ± 0.0918 | 1.59 ± 0.731 |
| ***LAMA1*** | 0.853 ± 0.189 | 0.627 ± 0.0737 |
| ***LAMA4*** | 0.154 ± 0.0614 | 1.53 ± 1.08 |
| ***LAMB2*** | 0.569 ± 0.025 | 1.01 ± 0.645 |
