# Supplementary material for: ERK5 Is Required for Tumor Growth and Maintenance Through Regulation of the Extracellular Matrix in Triple Negative Breast Cancer
Source: Front Oncol. 2020 Aug 3;10:1164. doi: 10.3389/fonc.2020.01164 (PMC7416559; doi:10.3389/fonc.2020.01164)
Supplement: Supplementary file 15 [file Table_2.docx]

**Supplementary Table II. Integrin gene expression changes in MDA-MB-231-ERK5*-*ko cells compared to parental controls.** Downregulated integrin genes identified from RNA sequencing of MDA-MB-231-ERK5*-*ko cells compared to parental controls. Integrin dimer partners and ligands associated with each gene are shown. Adapted from information found in Barczyk et *al.* (45) and LaFoya et *al.* (56).

| **Gene Name** | **Gene expression (fold change)** | **Integrin dimer partner(s)** | **Matrix and non-matrix ligands** |
| --- | --- | --- | --- |
| ***ITGA1*** | 0.181114 | β1 | Collagens I, IV, and IX |
| ***ITGA2*** | 0.104215 | β1 | Collagens I, IV, and IX |
| ***ITGA5*** | 0.169315 | β1 | Fibronectin, glycoprotein NMB (GPNMB), and ANGPTLs |
| ***ITGA6*** | 0.315025 | β1, β4 | Laminins 511, 332, 111, and 411 |
| ***ITGA10*** | 0.212156 | β1 | Collagens II, IV, VI, and IX |
| ***ITGA11*** | 0.297373 | β1 | Collagens I, IV, and IX |
| ***ITGB3*** | 0.33535 | αIIB, αV | Fibrinogen, fibronectin, vitronectin, L1CAM, ANGPTLs, pro-TGFβ, VEGF, trans-resveratrol, DHT, T3, T4, and Tetrac |
| ***ITGB7*** | 0.163684 | αE, α4 | E-cadherin, MadCAM-1, VCAM-1, and fibronectin |
